# Intracellular SCARB2 mimicry overcomes decoy failure to broadly inhibit enteroviruses

**DOI:** 10.1371/journal.ppat.1014699

**Published:** 2026-10-13

**Authors:** Shijin Wang, Yubin Tang, Songyang Lai, Jiaxin Yang, Qingran Yang, Xize Liu, Xiao-Fang Yu, Haoran Guo, Wei Wei

**Affiliations:** 1 Cancer Centre, The First Hospital of Jilin University, Changchun, Jilin, China; 2 Institute of Virology and AIDS Research, The First Hospital of Jilin University, Changchun, Jilin, China; 3 Department of Respiration, Children’s Medical Center, First Hospital, Jilin University, Changchun, Jilin, China; 4 Cancer Institute (Key Laboratory of Cancer Prevention and Intervention, China National Ministry of Education), The Second Affiliated Hospital, Zhejiang University School of Medicine, Hangzhou, Zhejiang China; 5 Institute of Translational Medicine, Key Laboratory of Organ Regeneration and Transplantation of Ministry of Education, The First Hospital of Jilin University, Changchun, Jilin, China; Stanford University, UNITED STATES OF AMERICA

## Abstract

Enterovirus A71 (EV-A71) and coxsackievirus A16 (CV-A16) are leading causes of severe hand, foot, and mouth disease (HFMD) with high childhood mortality and no specific antiviral therapy. SCARB2 is the essential uncoating receptor, yet extracellular SCARB2-Fc decoys fail to block infection, representing a decade-old paradox in enterovirus therapeutics. Here, we overcome this limitation by engineering RMP(R8), a cell-penetrating SCARB2 mimetic peptide comprising the minimal VP2-binding domain (W146-K166) fused to an octa-arginine transporter. RMP(R8) shows potent antiviral activity (EC_50_ = 0.156 μg/mL), outstanding safety (selectivity index > 2003), and excellent aqueous solubility, a critical asset for clinical translation. It broadly inhibits genetically divergent EV-A71 isolates and CV-A16 in intestinal and neural cells by blocking viral entry. In a lethal neonatal mouse model, RMP(R8) increases survival against EV-A71 and CV-A16 challenge *in vivo*. Intracellular receptor mimicry thus establishes a generalizable strategy for viruses refractory to extracellular intervention. RMP(R8) is a promising clinical candidate to address the unmet need for targeted anti-enterovirus treatment, and our findings redefine how to drug virus-receptor interactions that act inside cells.

## Author summary

Hand, foot, and mouth disease (HFMD) caused by EV-A71 and CV-A16 affects millions of children globally each year, but no targeted antiviral drugs exist. Although SCARB2 has long been known as the key uncoating receptor for these pathogens, extracellular soluble SCARB2 decoy strategies have uniformly failed to block infection. In this study, we solved this puzzle by delivering a tiny SCARB2 fragment inside cells instead of keeping it outside. The resulting peptide, RMP(R8), is highly potent, safe, soluble in water, and protects mice from lethal enterovirus infection. Importantly, this work establishes a new antiviral paradigm: for viruses that use receptors inside cells to invade, intracellular receptor mimicry works where traditional extracellular approaches fail. RMP(R8) offers a translatable drug candidate to treat severe HFMD, and our strategy can be adapted to fight many other hard-to-target viral pathogens.

## Introduction

Hand, foot, and mouth disease (HFMD) causes millions of infections annually worldwide, with enterovirus A71 (EV-A71) and coxsackievirus A16 (CV-A16) responsible for the majority of severe cases and childhood deaths in the Asia-Pacific region [1]. Despite the availability of inactivated EV-A71 vaccines, no specific antiviral therapy exists for infected children, leaving clinicians with only supportive care options during outbreaks [2,3]. The recurring epidemics and potential for neurological complications underscore an urgent need for effective antiviral strategies that can complement vaccination efforts and treat established infections [4,5].

Targeting virus-host receptor interactions represents one of the most validated approaches in antiviral drug discovery, exemplified by HIV entry inhibitors targeting CD4 and CCR5 [6–8]. For EV-A71, SCARB2 was identified as the functional receptor over a decade ago, mediating viral attachment and uncoating through direct binding to the viral capsid [9]. This discovery initially sparked considerable enthusiasm for developing SCARB2-based therapeutics [10], particularly soluble receptor decoys that could neutralize virus particles before cellular entry, a strategy that has succeeded against other pathogens including rhinoviruses (via ICAM-1-Fc fusion proteins) and coronaviruses (via ACE2-Fc fusion proteins) [11,12].

However, equivalent approaches targeting SCARB2, the well-characterized receptor for EV-A71, CV-A16, and related group A enteroviruses, have consistently failed, with SCARB2-Fc fusion proteins showing no significant antiviral activity [13,14]. This paradox suggests that the SCARB2-EV-A71 interaction is mechanistically distinct and that alternative therapeutic modalities may be required to exploit this host-virus dependency.

Structural studies further reveal that SCARB2 binds EV-A71 at the southern rim of the capsid canyon in an asymmetric manner, distinct from canonical receptors that insert deeply into the canyon [15–17]. Guided by this interface, we initially designed a proteolysis-targeting chimera (PROTAC)-like peptide derived from the VP2-binding region of SCARB2 to redirect viral components for intracellular degradation. Unexpectedly, we discovered that intracellular delivery alone conferred potent antiviral activity independent of the ubiquitin-proteasome system. The optimized candidate, RMP(R8), displays exceptional potency against genetically diverse EV-A71 clinical isolates, favorable aqueous solubility, an important asset for peptide translation, and broad-spectrum activity against the clinically relevant SCARB2-dependent virus CV-A16. Mechanistically, RMP(R8) acts by blocking viral entry, and *in vivo* studies demonstrate significant protection against lethal enterovirus infection. These findings establish intracellular receptor mimicry as a powerful and generalizable antiviral paradigm, especially for viruses whose unusual receptor engagement renders conventional extracellular approaches ineffective.

## Results

### Engineering of an intracellular EV-A71-targeting peptide based on the SCARB2-EV-A71 interaction interface

Characterization of viral receptors and virus-receptor interaction mechanisms has enabled the development of receptor-mimetic strategies that intercept virions extracellularly and block viral entry. This approach has been successfully applied to the enterovirus A (EV-A) receptor KREMEN1 (for coxsackievirus A10, CV-A10) [18] and our recently identified enterovirus D (EV-D) receptor MFSD6 (for EV-D68) [19,20]: soluble Fc-fusion proteins built on the receptor-virus critical interfaces potently inhibited EV-D68 and CV-A10 replication, respectively. However, this strategy failed when applied to SCARB2, the receptor for the EV-A member EV-A71: a similarly designed SCARB2-Fc fusion protein did not significantly affect EV-A71 replication.

In fact, despite the earlier discovery of SCARB2 relative to KREMEN1 and MFSD6, no SCARB2-derived antiviral strategy against EV-A71 has been established to date [1]. Nevertheless, structural biology has yielded a nearly complete understanding of the SCARB2-EV-A71 interaction, with the binding interface thoroughly resolved [15–17]. We therefore designed a receptor-mimetic peptide derived from the EV-A71-binding region of SCARB2 to capture virions intracellularly. Drawing on the PROTAC concept, we aimed to deliver viral particles or structural proteins to the intracellular VHL ubiquitination system for proteasomal degradation, thereby clearing viral components and exerting antiviral activity.

In contrast to canonical enterovirus receptors, which span the “canyon” surrounding the five-fold icosahedral axis of the viral capsid, insert deeply into the canyon floor, and contact conserved residues of VP1, VP2, and VP3, SCARB2 binds the southern rim of the canyon around the five-fold axis of EV-A71 in a unique asymmetric manner. SCARB2 engages EV-A71 via its α5 and α7 helices, which form specific interactions with the VP1 GH loop and VP2 EF loop of EV-A71 (Fig 1A). To avoid interference from viral entry dynamics and conformational plasticity of progeny virions, we selected a discrete SCARB2 subdomain that selectively recognizes VP2 (residues W146-K166) as the receptor-mimetic peptide (RMP) core (Fig 1A, 1B). We fused the canonical VHL-recognition motif ALAPYIP and a cell-penetrating peptide (either octa-arginine, R8, or TAT) at the C-terminus to construct two intracellular antiviral peptides, designated RMP_VHL_(R8) and RMP_VHL_(TAT) (Fig 1B).

**Fig 1:**
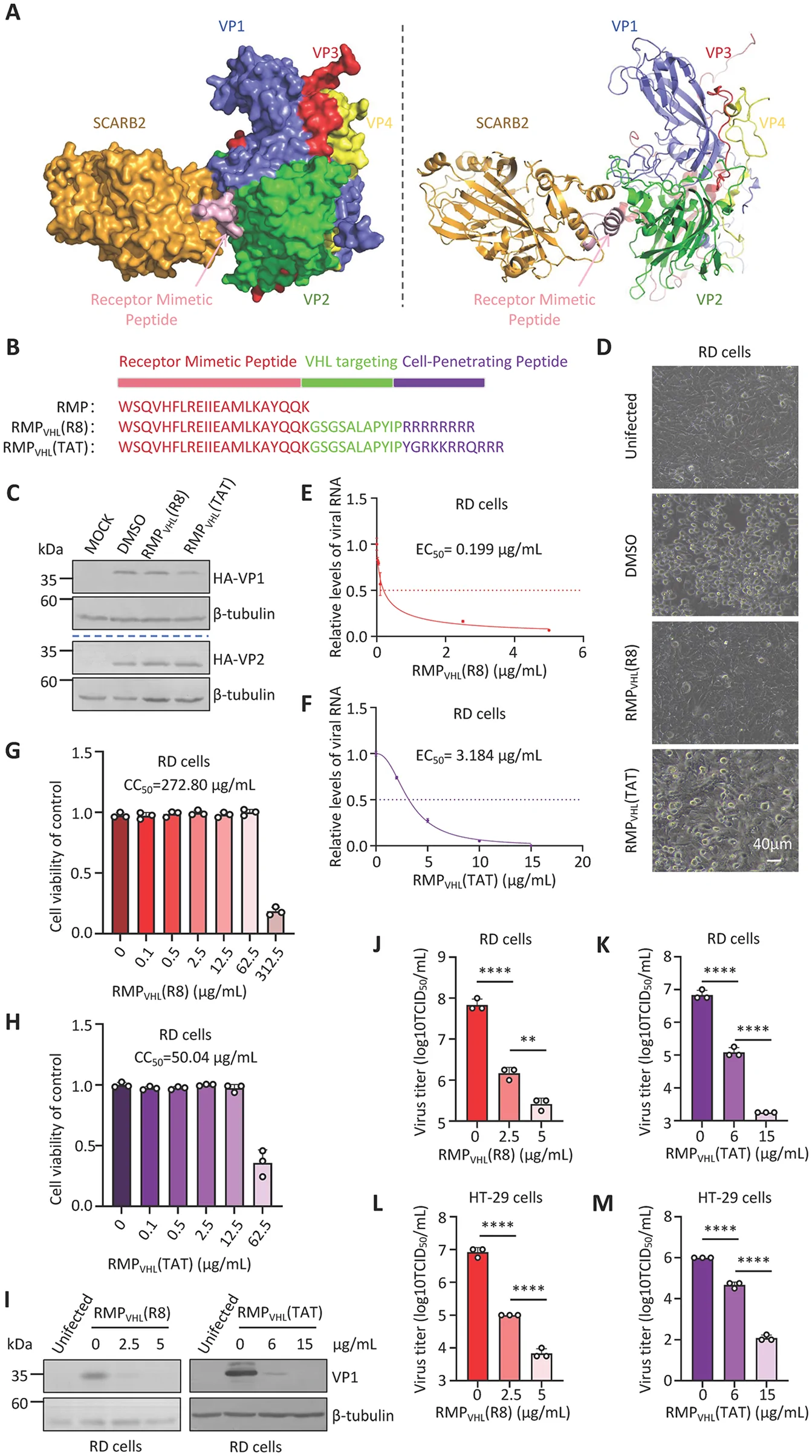
Identification of SCARB2-derived peptides with antiviral activity against EV-A71. **(A)** Structure of the EV-A71 asymmetric unit, with VP1 (light purple), VP2 (green), VP3 (red), VP4 (bright yellow), and the SCARB2-binding peptide (pink) located within the canyon region. **(B)** Schematic of PROTAC design based on a receptor-mimetic peptide. **(C)** For transfection-based experiments, HEK293T cells transfected with HA-tagged VP1 or VP2 expression constructs were treated with the indicated PROTACs (10 µg/mL) for 12 h, after which the cells were harvested for immunoblotting analysis. **(D)** CPE in EV-A71-infected RD cells (MOI = 0.01). For direct peptide treatment, EV-A71 was pre-incubated with PROTACs (5 µg/mL) at 37°C for 2 h prior to infection. Images were captured at 48 h post-infection. (Scale bar, 40 µm) **(E and F)** Dose-response curves and EC_50_ values for inhibition of EV-A71 RNA replication by RMP_VHL_(R8) (E) and RMP_VHL_(TAT) **(F)**. **(G and H)** Cytotoxicity of RMP_VHL_(R8) (G) and RMP_VHL_(TAT) (H) in RD cells, with CC_50_ values indicated. **(I-K)** RMP_VHL_(R8) and RMP_VHL_(TAT) inhibited viral protein expression (I) and viral titers (J and K) in EV-A71-infected RD cells (MOI = 0.01) following the standard pre-incubation protocol. **(L and M)** Similar reduction in viral titers by RMP_VHL_(R8) (L) and RMP_VHL_(TAT) (M) in EV-A71-infected HT-29 cells (MOI = 0.01). Error bars denote SD. **p < 0.01; ****p < 0.0001.

We next examined the degradation of the viral structural protein VP2 by RMP_VHL_(R8) and RMP_VHL_(TAT). Neither peptide significantly reduced VP2 levels; similarly, EV-A71 VP1 expression was unaffected (Fig 1C). Unexpectedly, despite the absence of PROTAC-like degradation activity, both peptides markedly inhibited EV-A71 replication and strongly suppressed virus-induced cytopathic effect (CPE) in cultured cells (Fig 1D). The half-maximal effective concentration (EC_50_) values were 0.199 μg/mL for RMP_VHL_(R8) and 3.184 μg/mL for RMP_VHL_(TAT) (Fig 1E, 1F). We ruled out non-specific cytotoxicity as a confounding factor (Fig 1G, 1H): the selectivity index (SI) reached 1370.85 for RMP_VHL_(R8) and 15.71 for RMP_VHL_(TAT), confirming specific inhibition of EV-A71 replication. Consistent with these findings, RMP_VHL_(R8) and RMP_VHL_(TAT) reduced intracellular accumulation of EV-A71 VP1 in a dose-dependent manner (Fig 1I). Moreover, the peptides inhibited EV-A71-induced CPE, viral protein expression, and progeny virion titers in a cell-type-independent manner in both human rhabdomyosarcoma (RD) and intestinal HT-29 cells (Fig 1J-1M; S1 Fig). These results led to the unexpected discovery of RMP_VHL_(R8), a highly potent SCARB2-derived antiviral peptide.

To elucidate why the PROTAC design failed to induce VP2 degradation despite potent antiviral activity, we performed co-immunoprecipitation (Co-IP) assays to test whether RMP_VHL_(R8) bridges VHL and VP2 into a functional ternary complex. Unexpectedly, HA-tagged EV-A71 VP2 constitutively co-immunoprecipitated with Flag-tagged VHL even in the absence of RMP_VHL_(R8) (S2 Fig). This indicates that VP2 possesses an intrinsic affinity for the VHL E3 ligase complex, independent of the chimeric peptide. Consequently, the failure of proteasomal degradation is not attributable to defective ternary complex formation or unfavorable spatial orientation for ubiquitin transfer. Instead, this pre-existing VP2-VHL association likely reflects a viral adaptation that renders VP2 tolerant to VHL-mediated ubiquitination.

### Intracellular delivery of SCARB2-derived RMP confers potent antiviral activity

We next investigated the molecular basis underlying the gain-of-antiviral-function of the previously inactive SCARB2 RMP. We hypothesized that intracellular delivery enables the RMP to inhibit viral replication within host cells. To test this hypothesis, we chemically synthesized the unmodified RMP and RMP fused only to the R8 cell-penetrating peptide (Fig 2A). Using FAM-labeled RMP(R8) and unmodified FAM-RMP as controls, we confirmed efficient cellular uptake and cytoplasmic localization exclusively for RMP(R8), whereas free RMP exhibited negligible intracellular fluorescence (Fig 2B). To define the subcellular compartment where RMP(R8) intercepts incoming virions, we performed triple-staining confocal immunofluorescence analysis of FAM-RMP(R8), EV-A71 VP1, and the late endosomal marker LAMP1 in infected RD cells. All three signals showed substantial overlap within the same LAMP1-positive compartments (S3 Fig). These results indicate that RMP(R8) and incoming virions converge within late endosomal compartments.

**Fig 2:**
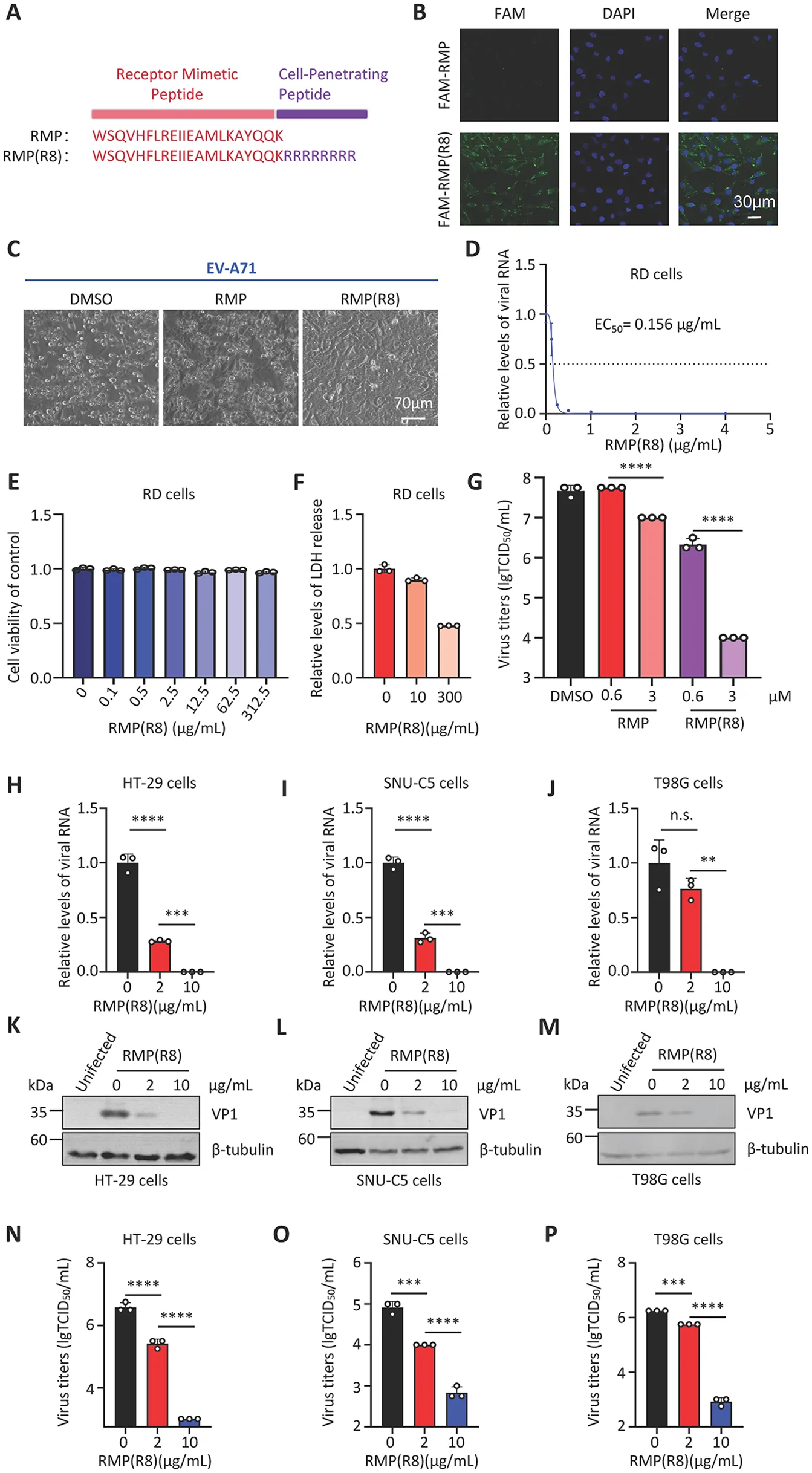
Cell-penetrating capability is essential for the antiviral activity of the SCARB2-derived peptide RMP(R8). **(A)** Schematic of peptides designed based on the receptor-mimetic peptide. **(B)** Immunofluorescence images of RD cells stained with FAM-labeled RMP(R8) (green) and DAPI (blue), showing exclusive cytoplasmic uptake of RMP(R8) but not unconjugated RMP. (Scale bar, 30 µm) **(C)** CPE in EV-A71-infected RD cells (MOI = 0.01) following pre-incubation of virus with indicated peptides (5 µg/mL) for 2 h prior to infection. Images were captured at 48 h post-infection. (Scale bar, 70 µm). **(D)** Dose-response curve and EC_50_ value for inhibition of EV-A71 RNA replication by RMP(R8). **(E)** Cytotoxicity profile of RMP(R8) in RD cells. **(F)** LDH release assay: RD cells were treated with RMP(R8) at indicated concentrations, relative LDH release was measured. **(G)** RMP(R8) inhibited viral titers in RD cells in a dose-dependent manner (MOI = 0.01). **(H-P)** RMP(R8) suppressed viral RNA levels(H-J), VP1 expression(K-M), viral titers(N-P) in HT-29 (MOI = 0.01), SNU-C5(MOI = 0.01), and T98G cells (MOI = 0.01). Error bars denote SD. n.s.,p > 0.05; *p < 0.05; **p < 0.01; ***p < 0.001; ****p < 0.0001.

Functional assays revealed that RMP(R8) exhibited even stronger antiviral activity than RMP_VHL_(R8), whereas the unfused RMP showed only modest inhibitory activity (Fig 2C, 2D). This weak activity of RMP was attributed to non-specific cellular uptake at higher concentrations, a phenomenon commonly observed for small peptides even without a dedicated cell-penetrating moiety. RMP(R8) showed an EC_50_ of 0.156 μg/mL and no significant cytotoxicity up to 312.5 μg/mL, yielding a SI of >2003 (Fig 2D, 2E). To further evaluate potential membrane-disrupting effects associated with cationic cell-penetrating peptides, we performed an LDH release assay. RMP(R8) did not induce significant LDH release at concentrations up to 300 μg/mL, confirming preservation of plasma membrane integrity (Fig 2F). In line with this, RMP(R8) potently reduced progeny virion titers, achieving a ~ 10^3^-fold inhibition of EV-A71 replication at 3 μM (Fig 2G).

### RMP(R8) Is a potent anti-EV-A71 peptide

To validate the broad antiviral activity of RMP(R8), we tested its efficacy in key EV-A71 target cells: intestinal epithelial lines HT-29 and SNU-C5, and the neural line T98G. RMP(R8) treatment robustly protected cells from EV-A71-induced CPE (S4 Fig), suppressed viral RNA replication (Fig 2H-2J), reduced viral protein expression (Fig 2K-2M), and lowered progeny virion release into culture supernatants (Fig 2N-2P). In contrast, unfused RMP showed negligible antiviral activity across these cell lines (S5 Fig). We further synthesized a scrambled control peptide fused to R8 (denoted R8) and confirmed that the R8 moiety itself does not confer antiviral activity (S6 Fig).

All preceding experiments employed the EV-A71 Anhui2007 strain [EV-A71(AH07)], isolated during the 2007 outbreak. To assess breadth against divergent EV-A71 isolates, we tested RMP(R8) against two clinically derived strains from Northeast China with distinct recombination patterns: EV-A71(CC063) and EV-A71(CC103). RMP(R8) significantly inhibited EV-A71-associated CPE (Fig 3A), viral RNA replication (Fig 3B-3E), viral protein expression (Fig 3F-3I), and progeny virion titers (Fig 3J-3M) for all tested strains. As a neurotropic enterovirus, EV-A71 infection of neural cells was also broadly suppressed by RMP(R8) (S7 Fig).

**Fig 3:**
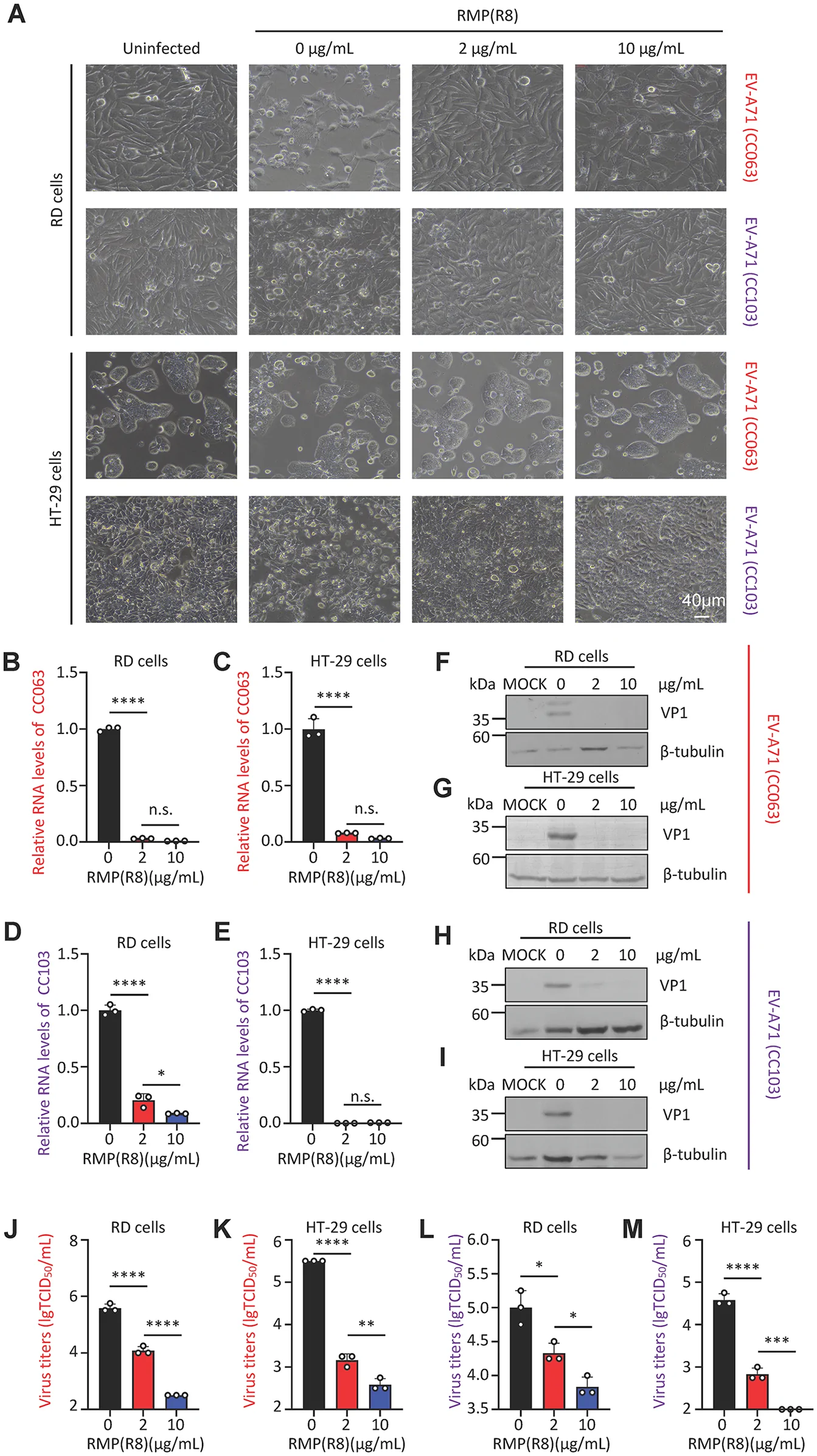
The SCARB2-derived peptide RMP(R8) exhibits broad-spectrum antiviral activity against contemporary EV-A71 strains. **(A-M)** EV-A71 CC063 (MOI = 0.01) and CC103 (MOI = 0.01) strains were pre-incubated with ultrapure water or different concentrations (2 and 10 µg/mL) of RMP(R8) for 2 h prior to infection of RD or HT-29 cells. CPE was recorded at 48 h post-infection.(Scale bar, 40 µm), and cells and supernatants were harvested for analysis. (B-I) RMP(R8) inhibited viral RNA levels (B-E) and VP1 protein expression (F-I) in both cell lines. **(J-M)** RMP(R8) reduced progeny virion titers in supernatants of cells infected with EV-A71 CC063 (J, K) and CC103 **(L, M)**. Error bars denote SD. n.s. p > 0.05; *p < 0.05; **p < 0.01; ***p < 0.001; ****p < 0.0001.

### RMP(R8) displays favorable aqueous solubility and targets EV-A71 entry

A favorable solubility profile is critical for clinical translation of peptide therapeutics. We compared the antiviral activity of RMP(R8) dissolved in DMSO [RMP(R8)-DMSO] versus ultrapure water [RMP(R8)-H_2_O]. RMP(R8)-H_2_O retained potent anti-EV-A71 activity comparable to RMP(R8)-DMSO across multiple assays (Fig 4A-4C).

**Fig 4:**
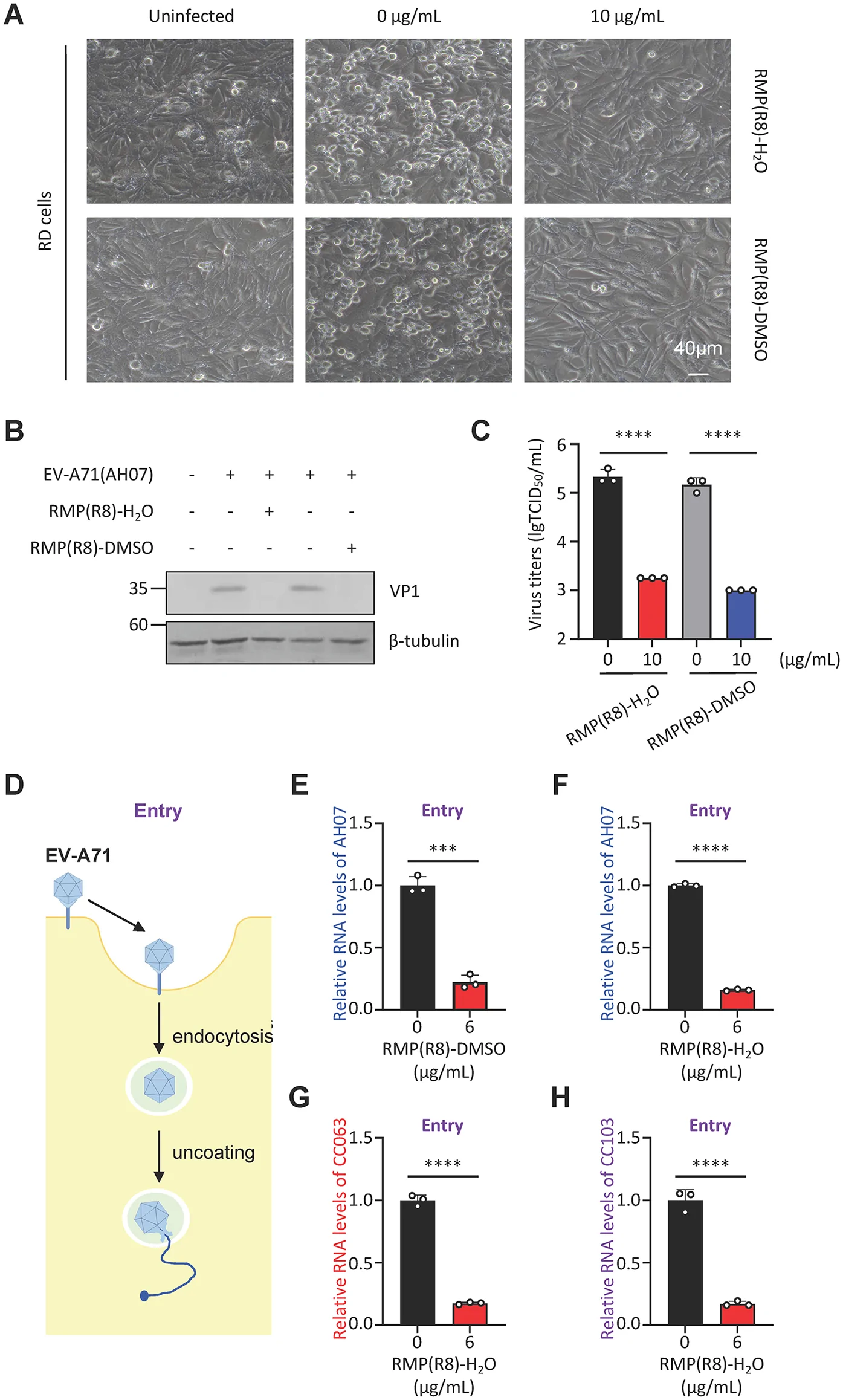
RMP(R8) is soluble in aqueous solution and primarily blocks EV-A71 entry. **(A-H)** EV-A71 viruses were pre-incubated with RMP(R8)-H_2_O, RMP(R8)-DMSO, H_2_O, or DMSO for 2 h prior to infection. At 48 h post-infection, CPE in RD cells was recorded (MOI = 0.01). Scale bar, 40 µm **(A)**. **(B and C)** Solvent variation does not affect the viral protein expression in cell lysates (B) and viral titers in supernatants **(C)**. **(D)** Schematic of the EV-A71 entry assay. **(E)** RMP(R8) dissolved in DMSO inhibits entry of EV-A71 strain AH07. **(F-H)** RMP(R8) dissolved in water inhibits entry of EV-A71 strains, including AH07 **(F)**, CC063 **(G)**, and CC103 **(H)**. For entry assays, virus-peptide mixtures were incubated at 4°C for 1 h followed by a temperature shift to 37°C for 0.5 **h.** Viral RNA was quantified by RT-qPCR. Error bars denote SD. ***p < 0.001; ****p < 0.0001.

SCARB2 acts as the primary entry receptor for EV-A71. To define the mechanism of action, we used a classical viral entry assay to examine the effect of RMP(R8) on the early stage of EV-A71 infection (Fig 4D). Both RMP(R8)-DMSO and RMP(R8)-H_2_O strongly inhibited EV-A71(AH07) entry (Fig 4E, 4F). Moreover, RMP(R8)-H_2_O significantly blocked entry of the divergent EV-A71 strains CC063 and CC103 (Fig 4G, 4H).

To definitively exclude direct neutralization or virucidal effects, we performed two complementary experiments. First, a viral pelleting assay showed that RMP(R8) does not alter the sedimentation profile of purified EV-A71 virions, with equivalent viral RNA recovered in the virion fraction regardless of peptide treatment, indicating that RMP(R8) does not disrupt capsid integrity or induce premature genome release (S8 Fig). Second, time-of-addition assays revealed that RMP(R8) retains maximal potency when added at 0–1 h post-infection, whereas its efficacy progressively declines at 1.5 hpi and is substantially diminished by 2 hpi (S9 Fig). This narrow temporal window is inconsistent with an irreversible extracellular virucidal mechanism and instead indicates that RMP(R8) acts at an early intracellular entry step.

To further evaluate its biopharmaceutical stability, RMP(R8) was incubated in mouse serum and simulated intestinal fluid at 37 °C, and residual antiviral activity was monitored over time. RMP(R8) exhibited a functional half-life of 14.17 h in mouse serum and 12.60 h in simulated intestinal fluid (S10 Fig), indicating favorable resistance to proteolytic degradation in both systemic and gastrointestinal environments. These stability profiles, together with its excellent aqueous solubility, support the translational potential of RMP(R8) as a candidate therapeutic peptide.

### Resistance profile of RMP(R8) under selective pressure

A major strength of targeting structurally constrained receptor-binding interfaces is the potential for a high barrier to resistance. To experimentally assess this, we performed serial passaging of EV-A71 in RD cells under continuous selective pressure with RMP(R8) (6 µg/mL) for eight consecutive passages (P8). A parallel control virus was passaged in the absence of peptide. Progeny virions harvested from passage 8 remained fully susceptible to RMP(R8), exhibiting equivalent suppression of viral VP1 expression, and infectious progeny titers upon re-challenge compared to the control-passaged virus (S11 Fig). These results suggest that the VP2-binding interface targeted by RMP(R8) is structurally constrained and functionally conserved, rendering it refractory to escape mutations under prolonged selective pressure.

### RMP(R8) protects mice from lethal EV-A71 challenge *in vivo*

To evaluate the *in vivo* antiviral efficacy of RMP(R8) against EV-A71, we employed a lethal neonatal mouse model using the neurovirulent EV-A71 CC063 strain. One-day-old ICR mice were inoculated intracranially with EV-A71 (1.0 × 10^7^ TCID_50_) that had been pre-incubated with RMP(R8) or vehicle control. In a parallel therapeutic regimen (post-treatment), mice were first inoculated intracranially with EV-A71 and subsequently administered a single intraperitoneal dose of RMP(R8) (10 mg/kg) or vehicle at 24 h post-infection. Over a 15-day observation period, the co-treatment group achieved 100% survival, whereas the vehicle control group succumbed uniformly (0% survival) (Fig 5A). Notably, even in the post-treatment setting, RMP(R8) conferred substantial protection, with survival rates significantly exceeding those of the control group (Fig 5B).

**Fig 5:**
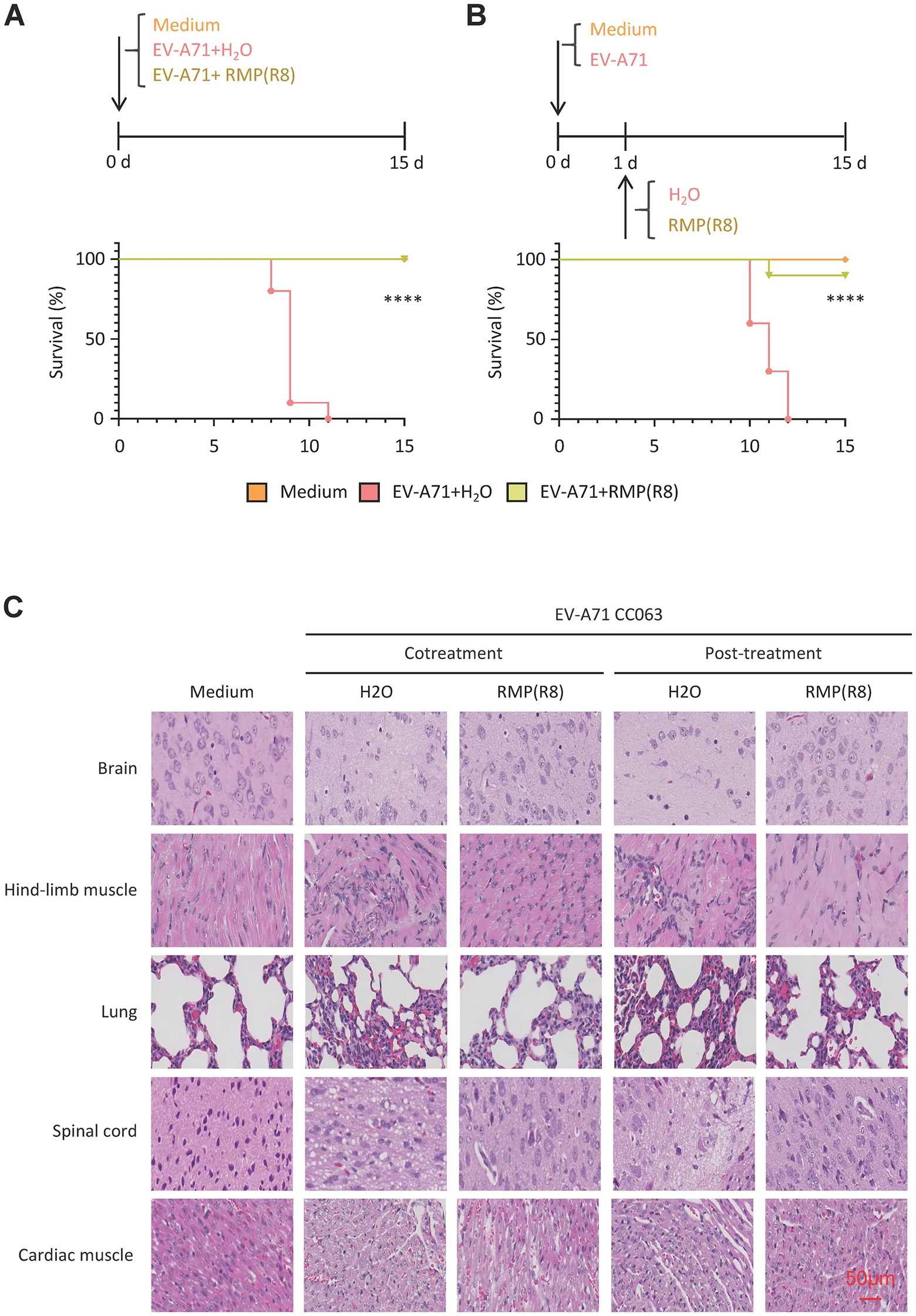
RMP(R8) protects neonatal mice from lethal EV-A71 challenge *in vivo.* **(A)** Survival curves of 1-day-old ICR mice inoculated intracranially with EV-A71 (1.0 × 10⁷ TCID_50_) under prophylactic (co-treatment) regimen: virus was pre-incubated with RMP(R8) or vehicle control for 30 min at 37°C prior to inoculation. **(B)** Survival curves under therapeutic (post-treatment) regimen: mice were first inoculated intracranially with EV-A71 and subsequently administered a single intraperitoneal dose of RMP(R8) (10 mg/kg) or vehicle at 24 h post-infection. Mice were monitored for 15 days post-infection. **(C)** Representative hematoxylin and eosin staining of brain, hind-limb muscle, lung, spinal cord, and cardiac muscle sections from vehicle- and RMP(R8)-treated mice at 7 days post-infection. Scale bars, 50 μm. Statistical significance was determined by log-rank (Mantel-Cox) test. ****p < 0.0001.

Histopathological analysis of major organs revealed severe lesions in the brains, hind-limb muscles, lungs, spinal cords, and cardiac muscles of vehicle-treated mice, characterized by inflammatory infiltration, necrosis, and tissue disorganization (Fig 5C). By contrast, RMP(R8)-treated mice displayed markedly preserved tissue architecture across all examined organs. Hence, RMP(R8) exerts potent protective efficacy against lethal EV-A71 infection *in vivo*.

### RMP(R8) effectively inhibits replication of the SCARB2-dependent virus coxsackievirus A16

In addition to EV-A71, coxsackievirus A16 (CV-A16), another major etiological agent of HFMD in children, also relies on SCARB2 for host cell entry [13]. We therefore evaluated the antiviral activity of RMP(R8) against CV-A16. A series of consistent results demonstrated that RMP(R8) effectively suppressed CV-A16-induced CPE across cell lines (Fig 6A), reduced viral RNA replication (Fig 6B-6D), and markedly diminished the production of infectious progeny virions in culture supernatants (Fig 6E-6G). Similar to EV-A71, RMP(R8) targeted the viral entry stage of CV-A16 infection (Fig 6H).

**Fig 6:**
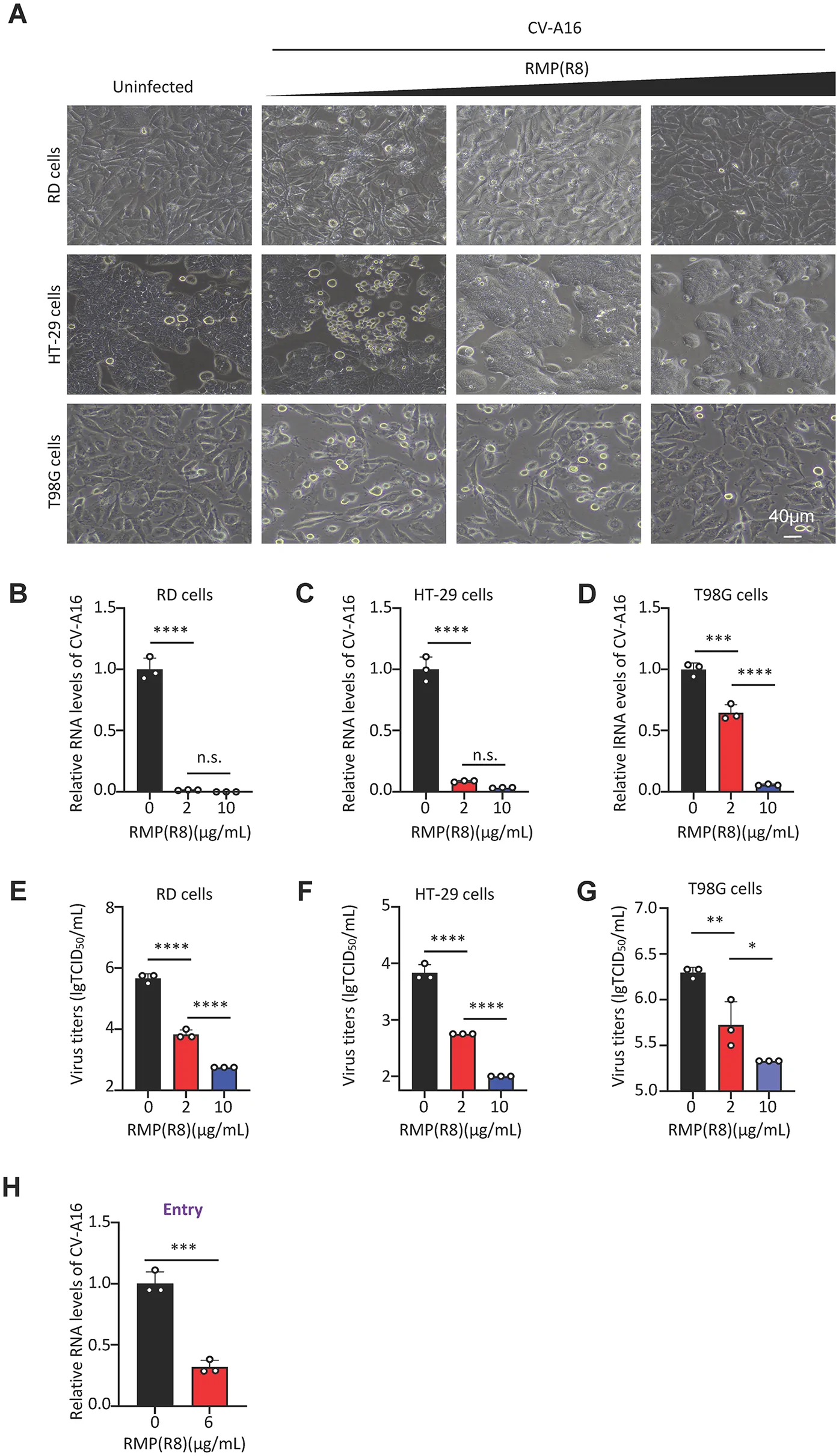
RMP(R8) inhibits coxsackievirus A16 (CV-A16) infection in vitro. **(A-H)** CV-A16 (MOI = 0.01) was pre-incubated with ultrapure water or increasing concentrations (2 and 10 μg/mL) of RMP(R8) at 37°C for 2 h prior to infection of RD, HT-29, and T98G cells. At 48 h post-infection, CPE was recorded (A; scale bar, 40 μm), viral RNA levels were quantified by RT-qPCR **(B-D)**, and progeny virion titers in supernatants were determined by TCID_50_ **(E-G)**. **(H)** CV-A16 entry assay (MOI = 2): virus-peptide mixtures were incubated at 4°C for 1 h followed by 37°C for 0.5 h, and viral RNA was quantified by RT-qPCR. Error bars denote SD. n.s. p > 0.05; *p < 0.05; **p < 0.01; ***p < 0.001; ****p < 0.0001.

### RMP(R8) protects mice from lethal CV-A16 challenge *in vivo*

We next evaluated the *in vivo* antiviral efficacy of RMP(R8) against CV-A16. In the co-treatment regimen, CV-A16 was pre-incubated with RMP(R8) or vehicle control prior to intraperitoneal inoculation of 1-day-old ICR mice. In a post-treatment paradigm, mice were first inoculated intraperitoneally with CV-A16 and subsequently administered a single intraperitoneal dose of RMP(R8) (10 mg/kg) or vehicle at 24 h post-infection. Over a 15-day observation period, the co-treatment group exhibited significantly improved survival compared to the vehicle control (0% survival for vehicle; Fig 7A). Notably, post-treatment administration of RMP(R8) also conferred substantial protective efficacy (Fig 7B).

**Fig 7:**
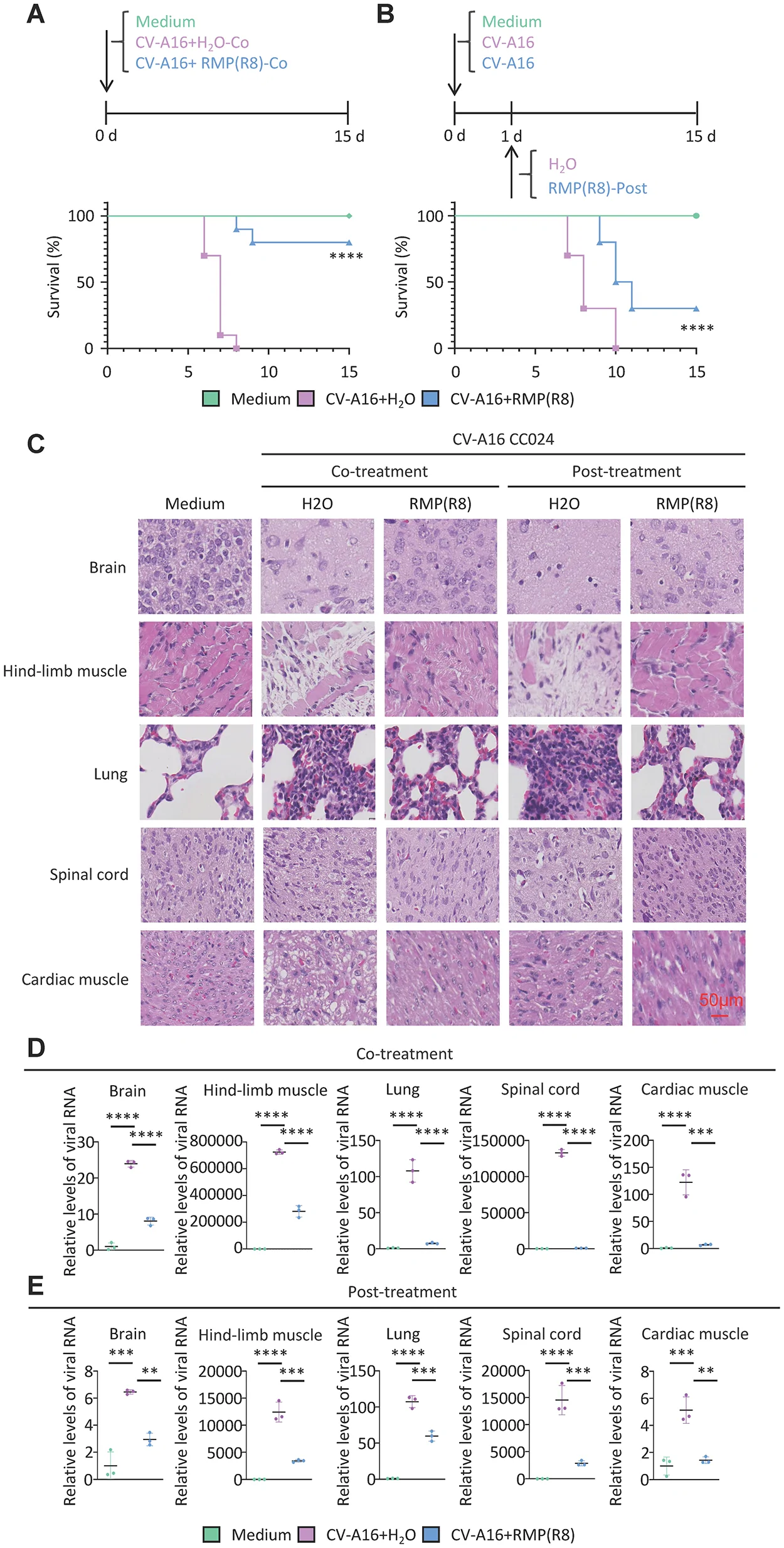
RMP(R8) protects mice from lethal CV-A16 challenge *in vivo.* **(A)** Survival curves of 1-day-old ICR mice inoculated intraperitoneally with CV-A16 (1.0 × 10⁷ TCID_50_) under prophylactic (co-treatment) regimen: virus was pre-incubated with RMP(R8) or vehicle control for 30 min at 37°C prior to inoculation. **(B)** Survival curves under therapeutic (post-treatment) regimen: mice were first inoculated intraperitoneally with CV-A16 and subsequently administered a single intraperitoneal dose of RMP(R8) (10 mg/kg) or vehicle at 24 h post-infection. Mice were monitored for 15 days post-infection. **(C)** Representative H&E staining of brain, hind-limb muscle, lung, spinal cord, and cardiac muscle sections from vehicle- and RMP(R8)-treated mice at 7 days post-infection. Scale bars, 50 μm. **(D, E)** Viral RNA loads in indicated tissues at 7 days post-infection under prophylactic (D) and therapeutic (E) regimens, quantified by RT-qPCR and normalized to GAPDH. Data are shown as mean ± SD. Statistical significance for survival curves was determined by log-rank (Mantel-Cox) test. *p < 0.05; **p < 0.01; ***p < 0.001; ****p < 0.0001.

We also harvested major target organs, including brain, hind-limb muscle, lung, spinal cord, and cardiac muscle for histopathological and virological analysis. Hematoxylin and eosin staining revealed severe pathological lesions in vehicle-treated mice (Fig 7C). In stark contrast, tissues from RMP(R8)-treated mice displayed substantially preserved architecture, with minimal inflammatory cell infiltration and absence of necrotic foci in both co-treatment and post-treatment cohorts (Fig 7C). Consistent with these, quantitative RT-PCR analysis demonstrated robust viral RNA accumulation across all examined organs in vehicle-treated mice. RMP(R8) treatment markedly suppressed viral loads in the brain, hind-limb muscle, lung, spinal cord, and cardiac muscle under both co-treatment and post-treatment conditions (Fig 7D, 7E). Collectively, these data demonstrate that RMP(R8) not only prolongs survival but also directly attenuates CV-A16 replication and mitigates virus-induced tissue injury *in vivo*, reinforcing its translational potential as a broad-spectrum therapeutic against SCARB2-dependent enteroviruses.

### RMP(R8) exhibits broad-spectrum activity against SCARB2-dependent enteroviruses

Phylogenetic analysis reveals that enterovirus A strains cluster into three major clades: one KREMEN1-dependent clade (including CV-A10), a SCARB2-dependent clade (including EV-A71 and CV-A16), and a third clade with unknown receptor usage [18,21,22] (Fig 8A). To test whether RMP(R8) exerts pan-clade activity across SCARB2-dependent enteroviruses, we evaluated its efficacy against coxsackievirus A7 (CV-A7) and coxsackievirus A14 (CV-A14), two additional members of this clade. RMP(R8) treatment significantly reduced infectious progeny virion titers of both CV-A7 and CV-A14 in a dose-dependent manner (Fig 8B, 8C). Concordantly, RMP(R8) potently protected cells from CV-A7- and CV-A14-induced cytopathic effects (Fig 8D). Collectively, these results confirm that RMP(R8) possesses broad-spectrum antiviral activity against genetically divergent SCARB2-dependent enteroviruses, establishing intracellular receptor mimicry as a generalizable strategy beyond individual viral species.

**Fig 8:**
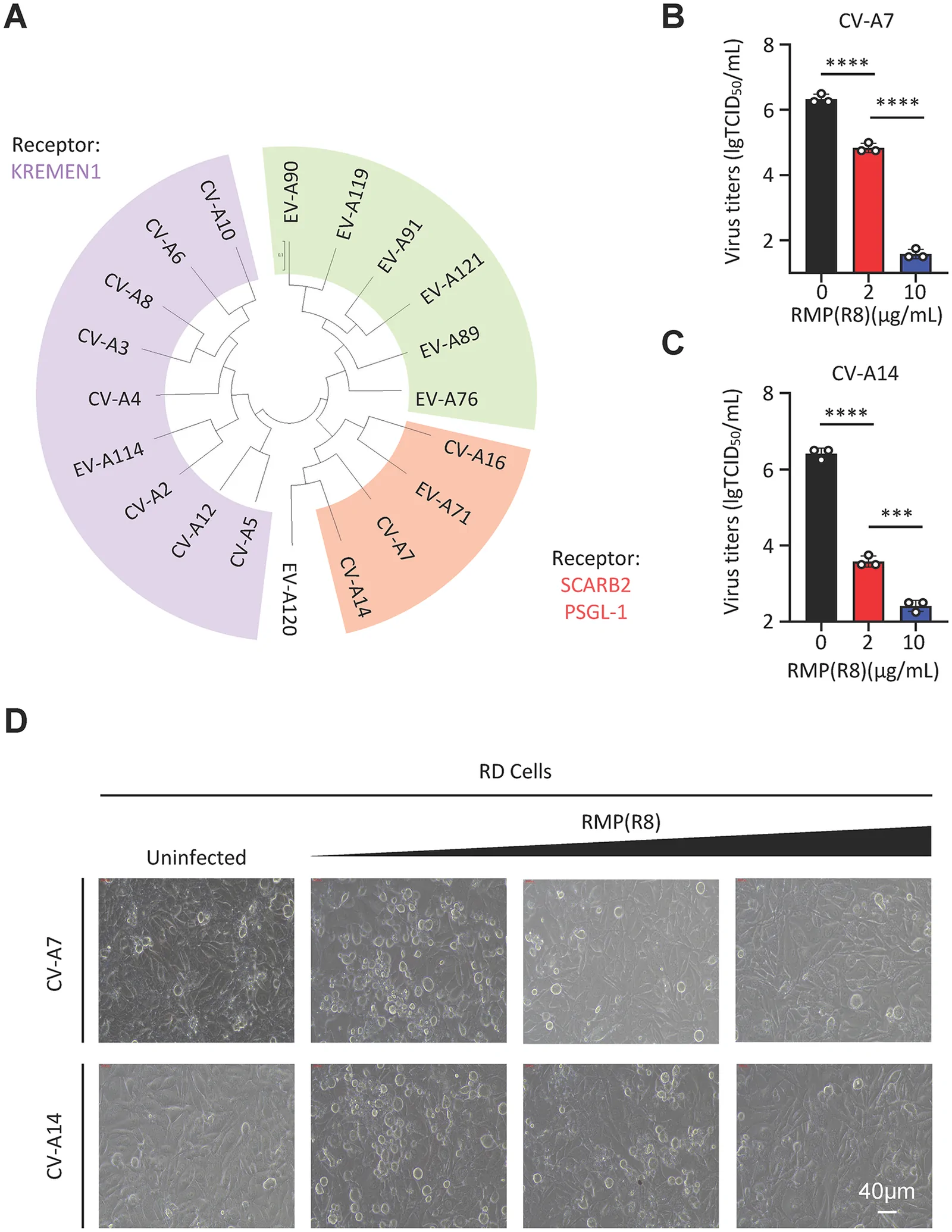
RMP(R8) exhibits broad-spectrum activity against SCARB2-dependent enteroviruses. **(A)** Phylogenetic analysis of enterovirus A strains showing three major clades: KREMEN1-dependent clade (including CV-A10), SCARB2-dependent clade (including EV-A71, CV-A16, CV-A7, and CV-A14), and a third clade with unknown receptor usage. **(B, C)** RD cells were infected with CV-A7 (B) or CV-A14 **(C)** (MOI = 0.01) following pre-incubation with increasing concentrations (2 and 10 μg/mL) of RMP(R8) for 2 h at 37°C. Progeny virion titers in supernatants were determined by TCID_50_ at 48 h post-infection. **(D)** Representative CPE images of RD cells infected with CV-A7 or CV-A14 (MOI = 0.01) following pre-incubation with RMP(R8) (10 μg/mL) or vehicle control. Images were captured at 48 h post-infection. Scale bar, 40 μm. Error bars denote SD. *p < 0.05; **p < 0.01; ***p < 0.001; ****p < 0.0001.

## Discussion

Hand, foot, and mouth disease caused by EV-A71 and CV-A16 remains a major threat to pediatric health, with no targeted therapeutics available to treat established infections [1]. Despite the identification of SCARB2 as the key receptor for these viruses over a decade ago, extracellular decoy strategies have consistently failed, creating a long-standing paradox in the field [22]. In this study, we address this unmet need by developing RMP(R8), a cell-penetrating receptor-mimetic peptide that achieves potent antiviral activity through intracellular intervention, overcoming the limitations of conventional SCARB2-targeted approaches.

Among enterovirus uncoating receptors, soluble receptor-Fc fusion proteins display significantly different antiviral activities. Specifically, KREMEN1-Fc effectively inhibits CV-A10 replication, and MFSD6-Fc effectively inhibits EV-D68 replication [18,23]. In contrast, at comparable protein concentrations, SCARB2-Fc shows almost no antiviral activity against EV-A71. Previous studies that reported soluble SCARB2-mediated EV-A71 uncoating and antiviral activity might be a result of non-physiological conditions, such as receptor overexpression systems or saturating protein concentrations [24,25]. This indicates that soluble SCARB2 can interfere with viral replication only under specific experimental contexts. In our side-by-side comparison with equivalent receptor-Fc doses, SCARB2-Fc demonstrated the lowest neutralizing efficiency, which implies fundamental differences in receptor recognition between the SCARB2-EV-A71 pair and other enterovirus-receptor pairs.

This observation aligns with the emerging understanding of EV-A71 receptor collaboration. PSGL-1 and SCARB2 represent two well-characterized functional receptors that cooperatively orchestrate viral entry [26]. PSGL-1, predominantly expressed on hematopoietic cells, promotes viral attachment to these cell lineages, while multiple cell surface molecules contribute to EV-A71 attachment across different cell types [27]. Critically, SCARB2 is the only known receptor that mediates viral uncoating following cell surface attachment [24,26]. This establishes a collaborative network where attachment receptors and uncoating receptors act in concert, with uncoating receptors exhibiting lower binding affinity than attachment receptors [28,29]. Indeed, SCARB2-Fc binds EV-A71 with significantly lower affinity than PSGL-1-Fc [24]. Structural studies provide a molecular explanation for this affinity difference: unlike canonical enterovirus receptors that insert deeply into the capsid canyon, SCARB2 engages the southern rim of the canyon region on the EV-A71 particle [15,30]. This unconventional binding mode likely contributes to the relatively low affinity between SCARB2 and EV-A71, partially explaining the difficulty in developing SCARB2-derived decoy receptors or peptides with direct antiviral activity.

Our initial strategy aimed to repurpose the SCARB2 virus-recognition domain through a PROTAC-like approach. We engineered RMP_VHL_(R8), a chimeric peptide designed to capture intracellular EV-A71 virions and recruit them to the VHL ubiquitin ligase complex for proteasomal degradation of viral structural proteins. However, viral protein analysis revealed that the peptide did not trigger degradation as intended. Co-immunoprecipitation assays unexpectedly demonstrated that EV-A71 VP2 constitutively associates with VHL even in the absence of RMP_VHL_(R8) (S1 Fig), indicating that the failure of proteasomal degradation is not due to defective ternary complex formation or unfavorable spatial orientation for ubiquitin transfer. Instead, this pre-existing VP2-VHL interaction likely reflects a viral adaptation to modulate the host ubiquitin-proteasome system during natural infection, rendering VP2 tolerant to VHL-mediated ubiquitination. This serendipitous observation led us to discover that simply conjugating the minimal RMP to a cell-penetrating peptide (R8) was sufficient to achieve even more potent antiviral activity (SI > 2003), effectively overcoming the long-standing limitations of SCARB2-based decoys (Fig 1, 2). Control experiments confirmed that neither the unconjugated RMP nor the R8 peptide alone possessed antiviral activity (S3-S5 Figs), further supporting our conclusion that the fusion of receptor fragment and cell-penetrating moiety is essential for efficacy.

The success of this cell-penetrating SCARB2 mimetic peptide offers a critical insight for SCARB2-based antiviral development: beyond the intrinsic binding affinity between SCARB2 and EV-A71, the spatiotemporal delivery of SCARB2-derived segments to the site of virus-receptor interaction may represent an equally decisive factor. We hypothesize that precise intracellular delivery of the SCARB2 fragment functionally overcomes the constraints imposed by the relatively low affinity of the SCARB2-EV-A71 interaction, thereby enabling effective blockade of viral replication. This interpretation is supported by our antiviral functional assays and by prior structural studies demonstrating that SCARB2 engages EV-A71 with lower affinity than canonical attachment receptors and through an unconventional binding geometry. Nevertheless, direct biophysical measurements (e.g., SPR or ITC) of RMP(R8) binding to VP2 or intact virions remain to be performed to rigorously test this model.

Another compelling aspect of this unexpectedly productive work is that our observations provide direct experimental support for the emerging view that SCARB2 does not encounter incoming virions primarily at the cell surface, but rather functions intracellularly to promote uncoating [14]. Because SCARB2 recognizes and facilitates viral uncoating predominantly within intracellular compartments rather than at the plasma membrane, targeted delivery of RMP to the intracellular milieu—rather than extracellular retention—maximizes its antiviral efficacy as a decoy receptor. Our findings thus validate the functional significance of landmark receptor identification and mechanism studies, demonstrating their translational potential. By achieving intracellular targeting of SCARB2-derived segments to the endogenous SCARB2-EV-A71 interaction site, we inhibit viral entry and uncoating, effectively turning the virus’s own receptor against itself.

RMP(R8) displayed several highly favorable properties that strengthen its translational potential. First, the peptide retained full antiviral activity when formulated in aqueous solution, eliminating reliance on organic solvents and overcoming a common limitation of peptide therapeutics. Second, RMP(R8) broadly inhibited genetically distinct EV-A71 clinical isolates with divergent recombination patterns, as well as infection of intestinal and neural cell lines that represent physiologically relevant target tissues. Third, RMP(R8) potently inhibited CV-A16, another major SCARB2-dependent HFMD pathogen, demonstrating activity across the clinically most relevant enterovirus group. Mechanistically, entry inhibition was consistently observed for both EV-A71 and CV-A16, supporting a unified mode of action that exploits shared SCARB2-dependent entry mechanisms. In a lethal neonatal mouse model, RMP(R8) increased survival against both EV-A71 and CV-A16, providing *in vivo* proof-of-concept that intracellular receptor mimics can function in authentic host environments. Collectively, these data position RMP(R8) as a promising candidate for further clinical development.

Our serial-passaging assay evaluated the potential emergence of RMP (R8)-resistant EV-A71 variants (S11 Fig). It should be noted that our resistance experiment used a single constant concentration of RMP(R8) rather than a stepwise-escalating selection scheme, which may provide relatively limited selection pressure and could underestimate the potential for resistance emergence. In addition, the viral populations were not sequenced; therefore, our conclusion is limited to the phenotypic observation that no resistant variant emerged under these conditions. Further studies using escalating drug concentrations and deep sequencing will be required to fully define the genetic barrier to RMP(R8) resistance.

The apparently narrow temporal window of RMP(R8) activity observed *in vitro* should be interpreted in the context of the experimental setting. In synchronized cell-culture infection, viral entry occurs within a short and relatively uniform time frame, so the peptide must be present during this early window to be effective (S9 Fig). *In vivo*, however, infection is asynchronous and multi-cycle, with ongoing viral spread to new cells and tissues at later time points. Thus, post-treatment administration at 24 h can still intercept subsequent rounds of virion entry and limit dissemination, even though it cannot reverse entry events that occurred before treatment (Fig 5). This distinction is consistent with the partial protection observed in the post-treatment groups and supports a model in which RMP(R8) acts by blocking new entry events throughout the course of infection rather than by neutralizing a single synchronized inoculum.

Beyond the specific findings for EV-A71 and CV-A16, this study advances the broader field of antiviral receptor targeting by redefining the principles for effective intervention. For many viruses, receptors have been dichotomously viewed as either “druggable” by extracellular decoys or “undruggable” when such approaches fail [31–34]. Our results demonstrate that receptor-derived molecules can be rendered effective simply by redirecting their site of action, even when conventional extracellular strategies prove ineffective. This principle is likely generalizable to numerous viruses that employ endosomal receptors, depend on intracellular uncoating, or engage their receptors through noncanonical orientations. In this sense, RMP(R8) represents not only a candidate therapeutic for HFMD but also a prototype for a platform strategy adaptable to other challenging viral pathogens.

In summary, this work resolves a decade-old paradox in SCARB2-targeted antiviral development by demonstrating that intracellular receptor mimicry overcomes the inherent limitations of extracellular decoys. RMP(R8), a cell-penetrating peptide derived from the minimal VP2-binding region of SCARB2, combines exceptional potency with aqueous solubility and broad-spectrum activity, positioning it as a promising clinical candidate against major HFMD pathogens. More broadly, this study establishes a paradigm-shifting antiviral strategy: for viruses whose unconventional receptor engagement renders conventional approaches ineffective, redirecting receptor fragments to the intracellular site of action can unlock potent therapeutic activity. In doing so, our findings not only validate SCARB2 as a mechanistically and therapeutically targetable receptor but also provide a generalizable blueprint for intercepting viral pathogens that evade traditional extracellular intervention.

## Materials and methods

### Ethics statement

All animal experiments in this study were reviewed and approved by the Institutional Animal Care and Use Committee of The First Hospital of Jilin University (Approval No: SYXK(JI)2022–0001). All animal manipulations complied with institutional animal welfare guidelines.

### Cells

HEK293T cells (human embryonic kidney cell lines, ATCC, CRL-3216), RD cells (human rhabdomyosarcoma cell lines, ATCC, CCL-136), T98G cells (human glioblastoma multiforme cell lines, ATCC, CRL-1690), SNU-C5 cells (human intestinal epithelial cell lines, Cell Bank of the Chinese Academy of Sciences, SCSP-5278), and HT-29 cells (human intestinal epithelial cell lines, Cell Bank of the Chinese Academy of Sciences, TCHu103) cells were used in this study. RD and T98G cells were cultured in Dulbecco’s modified Eagle’s medium (Pricella, PM150210), whereas SNU-C5 and HT-29 cells were maintained in RPMI 1640 medium (Pricella, PM150114). All culture media were supplemented with 10% fetal bovine serum (FBS) and 1% penicillin-streptomycin. Cells were cultured at 37°C in a humidified atmosphere containing 5% CO_2_. All cell lines were authenticated by the vendors and tested negative for mycoplasma contamination prior to experiments.

### Viruses

EV-A71 (CC063), EV-A71 (CC103), and CV-A16 (CC024) were stored in a -80°C freezer in our laboratory. EV-A71 (Anhui2007), CV-A7 (Parker strain), and CV-A14 (CHN_2014_YN_41 strain) were kindly provided by Drs. Tong Cheng, Chao Zhang, and Yong Zhang, respectively. Aliquots were thawed immediately prior to the experiments to avoid repeated freeze-thaw cycles. These viruses were amplified in RD cells. Infected cells were subjected to three freeze-thaw cycles, and supernatants were clarified by centrifugation at 3,000 rpm for 10 min, followed by filtration through a 0.45-µm filter. Viral particles were pelleted through a 20% sucrose cushion by ultracentrifugation in an SW41 Ti rotor (Beckman Coulter) at 28,000 rpm for 2 h at 4°C. Purified virions were resuspended and titrated by TCID_50_ assays in RD cells.

### Plasmids and reagents

VR1012-HA-EV-A71 VP1 and VR1012-HA-EV-A71 VP2 were constructed using the Hieff Clone Plus Multi One-Step Cloning Kit (Yeasen Biotech, 10912) with EV-A71 Anhui2007 as the template. The following antibodies were used: anti-beta tubulin antibody (1:2,000; HuaBio, M1305-2), anti-EV-A71 VP1 antibody (1:2,000; GeneTex, GTX132338), anti-Flag antibody (1:1000; Sigma-Aldrich, F1804), anti-HA antibody (1:2,000; Sigma-Aldrich, H9658), anti-EV-A71 VP1 antibody (1:1,000; GeneTex, GTX633390), anti-mouse Alexa 594 (1:1,000; Thermo Fisher Scientific, A-11005), anti-LAMP1 (D2D11#9091, Cell Signalling Technology) and anti-rabbit Alexa 647 (1:1000; Thermo Fisher Scientific, A-21245). All peptides, including RMP_VHL_(R8), RMP_VHL_(TAT), FAM-RMP(R8), RMP(R8), FAM-RMP, RMP, and R8, were synthesized by GenScript (Piscataway, NJ) and were obtained at purities exceeding 95%. The plasmid pcFlag-VHL_pcDNA3.4 (Genscript, C321MJBUG0–2/NY387407) was purchased from Genscript.

### Transfection and peptide treatment

HEK293T cells were seeded in 12-well plates and cultured for 24 h until reaching approximately 60% confluence. DNA transfection was performed using polyethylenimine (Polysciences, 24765) according to the manufacturer’s instructions. At 36 h post-transfection, cells were treated with the indicated peptides at the concentrations specified in the figure legends. Cells were harvested at 48 h post-transfection for immunoblot analysis.

### Co-immunoprecipitation (Co-IP)

HEK293T cells were transfected with HA-EV-A71 VP2 and/or Flag-VHL using polyethylenimine. At 36 h post-transfection, cells were treated with RMP_VHL_(R8) or vehicle for 12 h. Cells were then lysed in IP lysis buffer (50 mM Tris·HCl pH 7.4, 150 mM NaCl, 1% NP-40, 0.5% sodium deoxycholate, 1 mM EDTA) containing protease inhibitors for 30 min at 4 °C. Lysates were centrifuged at 13,000 rpm for 10 min, and supernatants were incubated with anti-HA magnetic beads (Roche, 11815016001) at 4 °C with gentle rotation. Beads were washed four times with IP lysis buffer and bound proteins were eluted for immunoblotting assay.

### Immunoblotting

Cells were harvested, washed twice with ice-cold PBS, and lysed in RIPA buffer (50 mM Tris·HCl pH 7.8, 150 mM NaCl, 1% NP-40, 5 mM EDTA) containing a protease inhibitor mixture for 30 min at 4°C. Lysates were mixed with 5 × SDS sample buffer (0.25 M Tris·HCl pH 6.8, 10% SDS, 50% glycerol, 0.5 M DTT, 0.5% bromophenol blue) and boiled at 100°C for 10 min. After centrifugation at 13,000 rpm for 10 min, equal amounts of protein were separated by SDS-PAGE and transferred to nitrocellulose membranes using a semidry apparatus (Bio-Rad). Membranes were blocked with 5% nonfat milk in TBST (20 mM Tris·HCl pH 7.6, 150 mM NaCl, 0.1% Tween-20) for 1 h, followed by overnight incubation at 4°C with primary antibodies. After washing, membranes were incubated with alkaline phosphatase-conjugated secondary antibodies [anti-rabbit (Jackson ImmunoResearch Laboratories, 111-035-045) or anti-mouse IgG (Jackson ImmunoResearch Laboratories, 115055–062)] for 1 h. Staining was conducted with 5-bromo-4-chloro-3-indolyl phosphate and nitrotetrazolium blue chloride solutions prepared from chemicals obtained from Sigma-Aldrich.

### Antiviral peptide treatment assays (infection-based)

For all peptide antiviral assays (unless otherwise specified), EV-A71 or CV-A16 was pre-incubated with the indicated peptide or vehicle control (DMSO or ultrapure water) for 2 h prior to infection. Cells were infected at the indicated multiplicity of infection (MOI) and cultured for 48 h before analysis.

### Viral entry assay

RD cells were incubated with peptide and virus (MOI of 2) at 4°C for 1 h, followed by a temperature shift to 37°C for 0.5 h to permit virus entry. After incubation, cells were washed twice with pre-warmed PBS. Total RNA was extracted, and viral RNA levels were quantified by RT-qPCR as described above.

### Viral titer assay

Viral titers were determined by microtitration analysis based on CPE in RD cells using the Reed-Muench method. Briefly, RD cells were seeded in 96-well plates at a density of 1 × 10⁴ cells per well and cultured overnight. EV-A71, CV-A16, CV-A7 or CV-A14 was serially diluted 10-fold in DMEM containing 2% FBS and added to the cells. After 72 h of incubation, CPE was recorded, and viral titers were calculated as the 50% tissue culture infectious dose (TCID_50_) per milliliter.

### Real-time quantitative reverse-transcription PCR (RT-qPCR)

Total RNA was extracted from cells or viral samples using TRIzol reagent (Thermo Fisher Scientific, 15596026) according to the manufacturer’s instructions. cDNA was synthesized from 1 µg of total RNA using Hifair III 1st Strand cDNA Synthesis SuperMix (Yeasen Biotech, 11141ES60). Real-time PCR was performed on an SLAN-96S real-time PCR system (Hongshi, China) using Hieff qPCR SYBR Green Master Mix (Yeasen Biotech, 11201ES03). The thermal cycling conditions were as follows: 95°C for 5 min; 40 cycles of 95°C for 10 s, 60°C for 20 s, and 72°C for 20 s. Melting curve analysis was performed to verify amplicon specificity. The following primer sequences were used: GAPDH forward, 5’-CCCACTCCTCCACCTTTGACG-3’; GAPDH reverse, 5’-CACCACCCTGTTGCTGTAGCCA-3’; EV-A71 forward, 5’-ACATGGTGTGAAGAGTCTATTGAGCT-3’; EV-A71 reverse, 5’-CCAAAGTAGTCGGTTCCGC-3’; CV-A16 forward, 5’-GTTATCCCACCTTCGGAGA-3’; CV-A16 reverse, 5’-TCGGGCATTGACCATAATCTAG-3’. Relative gene expression was calculated using the 2^-ΔΔCt^ method, with GAPDH serving as the internal control.

### Cell viability assay

Cells were seeded in 96-well plates at a density of 1 × 10^4^ cells per well and cultured until reaching 90% confluence. The medium was then replaced with fresh complete medium containing DMSO (vehicle control) or increasing concentrations of RMP_VHL_(R8), RMP_VHL_(TAT), and RMP(R8). After 48h of incubation, 10 µL of CCK-8 solution (MCE, HY-K0301) was added to each well and incubated for an additional 1 h at 37°C. Absorbance was measured at 450 nm using a BioTek microplate reader. Each experiment was performed in triplicate, and data were normalized to DMSO-treated control wells.

LDH release assay: RD cells were treated with increasing concentrations of RMP(R8) in serum-free DMEM for 48 h. LDH release into the culture supernatant was measured using an LDH Cytotoxicity Assay Kit (Beyotime, C0016) according to the manufacturer’s instructions. Absorbance was measured at 490 nm using a BioTek microplate reader.

### Immunofluorescence

Prior to addition of the fluorescent peptide, RD cells were washed twice with PBS and then incubated with 5 µg/mL FAM-RMP(R8) for 3 h. The cells were then washed twice with PBS, fixed with 4% paraformaldehyde for 30 min, and blocked with 5% BSA for 1 h. Nuclei were counterstained with 4’,6-diamidino-2-phenylindole (F6057, Sigma-Aldrich). Fluorescence imaging was performed using a confocal microscope (Nikon AX) equipped with 100 × oil objective. Images were acquired using NIS-Elements AR 5.41.01.

### Viral pelleting assay

Purified EV-A71 virions (1.0 × 10^7^ TCID_50_) were incubated with RMP(R8) or ultrapure water for 2 h. Samples were layered onto a 20% sucrose cushion in ultracentrifuge tubes and centrifuged at 28,000 rpm for 2 h at 4 °C using an SW41 Ti rotor (Beckman Coulter). Following centrifugation, fractions were collected from top to bottom as F1 (culture medium), F2 (20% sucrose interface), and F3 (virion pellet). Viral RNA in each fraction was extracted using TRIzol and quantified by RT-qPCR.

### Neonatal mouse infection studies

One-day-old ICR mice were obtained from Beijing Vital River Laboratory Animal Technology Co., Ltd. The mice were housed in individually ventilated cages under specific pathogen-free conditions. Newborn mice of approximately equal sex ratios were used for viral infectivity experiments.

EV-A71 challenge. For co-treatment, RMP(R8) or vehicle was incubated with EV-A71 CC063 (1.0 × 10^7^ TCID_50_) for 30 min prior to intracranial inoculation. For post-treatment, mice were first inoculated intracranially with EV-A71, followed by a single administration of RMP(R8) (10 mg/kg) or vehicle at 24 h post-infection. Mice were monitored daily for survival for 15 days. At 7 days post-infection, tissues (brain, hind-limb muscle, lung, spinal cord, cardiac muscle) were harvested for histopathological analysis. This protocol was used for experiments shown in Fig 5.

CV-A16 challenge. For co-treatment, RMP(R8) (10 mg/kg) or vehicle was incubated with CV-A16 CC024 (1.0 × 10⁷ TCID50) for 30 min prior to intraperitoneal inoculation of 1-day-old ICR mice. For post-treatment, mice were first inoculated intraperitoneally with CV-A16, followed by a single administration of RMP(R8) (10 mg/kg) or vehicle at 24 h post-infection. Mice were monitored daily for survival for 15 days. At 7 days post-infection, tissues were harvested for histopathological analysis and viral RNA quantification by RT-qPCR. This protocol was used for experiments shown in Fig 7.

### Statistics

Statistical data were analyzed using GraphPad Prism 8.0.2. Statistical analyses were performed via student’s t test and one-way analysis of variance (ANOVA). For the animal survival study, Kaplan-Meier survival curve was generated and analyzed by Log-Rank test. Statistical significance was assigned when P values were < 0.05. Error bars show mean and standard deviation (mean ± SD). The details of the statistical analyses were depicted in the figure legends.

## Supporting information

S1 Fig The SCARB2-derived peptide RMP_VHL_(R8) inhibits EV-A71 replication in intestinal epithelial cells. (A-C) Infection-based antiviral assay: EV-A71 (MOI = 0.01) was pre-incubated with DMSO or different concentrations of RMP_VHL_(R8) and RMP_VHL_(TAT) for 2 h prior to infection of HT-29 cells. CPE was recorded at 48 h post-infection. Scale bar, 40 µm. (B and C) Immunoblot analysis of VP1 expression in HT-29 cells treated with RMP_VHL_(R8) (B) and RMP_VHL_(TAT) (C).
(TIFF)

S2 Fig Interaction between EV-A71 VP2 and VHL. For transfection-based experiments, HA-tagged VP2 or empty vector was cotransfected with Flag-tagged VHL into HEK293T cells, then the cells were treated with RMPVHL(R8) (10 μg/mL) or vehicle control for 12 h. Cell lysates were immunoprecipitated (IP) with anti-HA antibody, followed by immunoblotting (IB) with anti-VHL and anti-HA antibodies.
(TIFF)

S3 Fig RMP(R8) colocalizes with EV-A71 VP1 and LAMP1-positive compartments. Confocal colocalization assay under infection conditions: RD cells were infected with EV-A71 in the presence of 5 µg/mL FAM-RMP(R8) for 3 h, then fixed and stained for EV-A71 VP1 (red) and the late endosomal marker LAMP1 (magenta). Nuclei were counterstained with DAPI (blue). Scale bar, 10 µm.
(TIFF)

S4 Fig RMP(R8) alleviates EV-A71-induced cytopathic effects across multiple cell types. Infection-based antiviral assay: EV-A71 AH07 (MOI = 0.01) strain was pre-incubated with ultrapure water or RMP(R8) (2 or 10 µg/mL) for 2 h prior to infection. (A) At 48 h post-infection, CPEs in HT-29, SNU-C5, and T98G cells were recorded (Scale bar, 40 µm).
(TIFF)

S5 Fig RMP without cell-penetrating peptide lacks antiviral activity against EV-A71. Infection-based antiviral assay: EV-A71 (MOI = 0.01) was pre-incubated with DMSO or RMP (2 or 10 µg/mL) for 2 h prior to infection of T98G or SNU-C5 cells. (A) CPE was recorded at 48 h post-infection. Scale bar, 40 µm. (B and C) Immunoblot analysis of VP1 expression in T98G (B) and SNU-C5 (C) cells. (D and E) Viral RNA levels in T98G (D) and SNU-C5 (E) cells. Error bars denote SD. “n.s.”, p > 0.05; **p < 0.01.
(TIFF)

S6 Fig Both the SCARB2-binding domain and cell-penetrating peptide are indispensable for RMP(R8) antiviral activity. (A-D) Infection-based antiviral assay: EV-A71(MOI = 0.01) was pre-incubated with DMSO, RMP(R8), RMP, R8 for 2 h prior to infection. (A) At 48 h post-infection of RD cells. (A) CPE at 48 h post-infection. Scale bar, 40 µm. (B) Immunoblot analysis of VP1 expression. (C) Viral RNA levels. (D) Viral titers in supernatants. Error bars denote SD. “n.s.”, p > 0.05, **p < 0.01; ****p < 0.0001.
(TIFF)

S7 Fig RMP(R8) inhibits replication of contemporary EV-A71 epidemic strains in neuronal T98G cells. (A) Infection-based antiviral assay: EV-A71 CC063 and CC103 (MOI = 0.01) were pre-incubated with ultrapure water or RMP(R8) (2 or 10 µg/mL) for 2 h prior to infection of T98G cells. CPE was recorded at 48 h post-infection. Scale bar, 40 µm. (B and C) Viral RNA levels of CC063 (B) and CC103 (C). (D and E) Immunoblot analysis of VP1 expression for CC063 (D) and CC103 (E). (F and G) Viral titers of CC063 (F) and CC103 (G). Error bars denote SD. “n.s.”, p > 0.05, *p < 0.05; **p < 0.01; ***p < 0.001; ****p < 0.0001.
(TIFF)

S8 Fig RMP(R8) does not disrupt the structural integrity of EV-A71 virions. Schematic of the viral pelleting assay. Purified EV-A71 virions were incubated with RMP(R8) (10 µg/mL) or H_2_O for 2 h, then ultracentrifuged through a 20% sucrose cushion (28,000 rpm, 2 h, 4 °C). Fractions were collected as F1 (culture medium), F2 (20% sucrose interface), and F3 (virion pellet). (B) EV-A71 RNA levels in each fraction determined by RT-qPCR. (C) Percentage distribution of EV-A71 RNA across fractions. Error bars denote SD.
(TIFF)

S9 Fig RMP(R8) acts during an early post-infection time window. Time-of-addition assay: RD cells were infected with EV-A71 (MOI = 0.01). RMP(R8) (10 µg/mL) was added at 0, 0.5, 1, 1.5, or 2 h post-infection (hpi). (A) Relative viral RNA levels at 48 hpi. (B) Viral titers in culture supernatants at 48 hpi. Error bars denote SD. ****P < 0.0001.
(TIFF)

S10 Fig RMP(R8) exhibits favorable metabolic stability in mouse serum and simulated intestinal fluids. RMP(R8) was incubated in mouse serum (A) or simulated intestinal fluids (B) at 37 °C for the indicated times. Residual antiviral activity against EV-A71 was determined by TCID_50_ assay in RD cells. Functional half-lives (t1/2) were calculated by nonlinear regression. Error bars denote SD.
(TIFF)

S11 Fig RMP(R8) does not readily select for resistant variants upon serial passaging. (A) Schematic of the serial passaging experiment. EV-A71 was passaged in RD cells in the presence of RMP(R8) (6 µg/mL) or H_2_O for eight passages (P1-P8). (B) CPE in RD cells infected with P8 viral stocks in the presence or absence of RMP(R8). Scale bar, 40 µm. (C) Immunoblot analysis of EV-A71 VP1 in cells infected with P0, P8[H_2_O] or P8[RMP(R8)] virus and treated with H_2_O or RMP(R8). (D-F) Viral titers of P0 (D), P8[H_2_O] (E) and P8[RMP(R8)] (F) viruses upon RMP(R8) treatment. Error bars denote SD. ****P < 0.0001; ***P < 0.001.
(TIFF)

S12 File Raw Gel. The raw data of western blot in this study. (PDF)

S13 Data The raw data supporting each of the manuscript Figures are contained in this Excel file. (XLSX)

## References

[1] Solomon T, Lewthwaite P, Perera D, Cardosa MJ, McMinn P, Ooi MH. Virology, epidemiology, pathogenesis, and control of enterovirus 71. Lancet Infect Dis. 2010;10(11):778–90. doi: 10.1016/S1473-3099(10)70194-8 20961813

[2] Li R, Liu L, Mo Z, Wang X, Xia J, Liang Z, et al. An inactivated enterovirus 71 vaccine in healthy children. N Engl J Med. 2014;370(9):829–37. doi: 10.1056/NEJMoa1303224 24571755

[3] Zhu F, Xu W, Xia J, Liang Z, Liu Y, Zhang X, et al. Efficacy, safety, and immunogenicity of an enterovirus 71 vaccine in China. N Engl J Med. 2014;370(9):818–28. doi: 10.1056/NEJMoa1304923 24571754

[4] Huang CC, Liu CC, Chang YC, Chen CY, Wang ST, Yeh TF. Neurologic complications in children with enterovirus 71 infection. N Engl J Med. 1999;341(13):936–42. doi: 10.1056/NEJM199909233411302 10498488

[5] Ooi MH, Wong SC, Lewthwaite P, Cardosa MJ, Solomon T. Clinical features, diagnosis, and management of enterovirus 71. Lancet Neurol. 2010;9(11):1097–105. doi: 10.1016/S1474-4422(10)70209-X 20965438

[6] Jacobson JM, Kuritzkes DR, Godofsky E, DeJesus E, Larson JA, Weinheimer SP, et al. Safety, pharmacokinetics, and antiretroviral activity of multiple doses of ibalizumab (formerly TNX-355), an anti-CD4 monoclonal antibody, in human immunodeficiency virus type 1-infected adults. Antimicrob Agents Chemother. 2009;53(2):450–7. doi: 10.1128/AAC.00942-08 19015347 PMC2630626

[7] Dalgleish AG, Beverley PC, Clapham PR, Crawford DH, Greaves MF, Weiss RA. The CD4 (T4) antigen is an essential component of the receptor for the AIDS retrovirus. Nature. 1984;312(5996):763–7. doi: 10.1038/312763a0 6096719

[8] Gulick RM, Lalezari J, Goodrich J, Clumeck N, DeJesus E, Horban A, et al. Maraviroc for previously treated patients with R5 HIV-1 infection. N Engl J Med. 2008;359(14):1429–41. doi: 10.1056/NEJMoa0803152 18832244 PMC3078519

[9] Yamayoshi S, Yamashita Y, Li J, Hanagata N, Minowa T, Takemura T, et al. Scavenger receptor B2 is a cellular receptor for enterovirus 71. Nat Med. 2009;15(7):798–801. doi: 10.1038/nm.1992 19543282

[10] Miwatashi W, Ishida M, Takashino A, Kobayashi K, Yamaguchi M, Shitara H, et al. Mouse Scarb2 Modulates EV-A71 Pathogenicity in Neonatal Mice. J Virol. 2022;96(15):e0056122. doi: 10.1128/jvi.00561-22 35867561 PMC9364792

[11] Alfaleh MA, Alsulaiman RM, Almahboub SA, Nezamuldeen L, Zawawi A, Aljehani ND, et al. ACE2-Fc and DPP4-Fc decoy receptors against SARS-CoV-2 and MERS-CoV variants: a quick therapeutic option for current and future coronaviruses outbreaks. Antib Ther. 2023;7(1):53–66. doi: 10.1093/abt/tbad030 38371953 PMC10873275

[12] Whiteman SC, Spiteri MA. IFN-gamma regulation of ICAM-1 receptors in bronchial epithelial cells: soluble ICAM-1 release inhibits human rhinovirus infection. J Inflamm (Lond). 2008;5:8. doi: 10.1186/1476-9255-5-8 18534017 PMC2427029

[13] Yamayoshi S, Iizuka S, Yamashita T, Minagawa H, Mizuta K, Okamoto M, et al. Human SCARB2-dependent infection by coxsackievirus A7, A14, and A16 and enterovirus 71. J Virol. 2012;86(10):5686–96. doi: 10.1128/JVI.00020-12 22438546 PMC3347270

[14] Nishimura Y, Sato K, Koyanagi Y, Wakita T, Muramatsu M, Shimizu H, et al. Enterovirus A71 does not meet the uncoating receptor SCARB2 at the cell surface. PLoS Pathog. 2024;20(2):e1012022. doi: 10.1371/journal.ppat.1012022 38359079 PMC10901359

[15] Zhou D, Zhao Y, Kotecha A, Fry EE, Kelly JT, Wang X, et al. Unexpected mode of engagement between enterovirus 71 and its receptor SCARB2. Nat Microbiol. 2019;4(3):414–9. doi: 10.1038/s41564-018-0319-z 30531980

[16] Dang M, Wang X, Wang Q, Wang Y, Lin J, Sun Y, et al. Molecular mechanism of SCARB2-mediated attachment and uncoating of EV71. Protein Cell. 2014;5(9):692–703. doi: 10.1007/s13238-014-0087-3 24986489 PMC4145081

[17] Yamayoshi S, Koike S. Identification of a human SCARB2 region that is important for enterovirus 71 binding and infection. J Virol. 2011;85(10):4937–46. doi: 10.1128/JVI.02358-10 21389126 PMC3126200

[18] Staring J, van den Hengel LG, Raaben M, Blomen VA, Carette JE, Brummelkamp TR. KREMEN1 Is a Host Entry Receptor for a Major Group of Enteroviruses. Cell Host Microbe. 2018;23(5):636-643.e5. doi: 10.1016/j.chom.2018.03.019 29681460

[19] Liu X, Li H, Li Z, Gao D, Zhou J, Ni F, et al. MFSD6 is an entry receptor for respiratory enterovirus D68. Cell Host Microbe. 2025;33(2):267-278.e4. doi: 10.1016/j.chom.2024.12.015 39798568

[20] Varanese L, Xu L, Peters CE, Pintilie G, Roberts DS, Raj S, et al. MFSD6 is an entry receptor for enterovirus D68. Nature. 2025;641(8065):1268–75. doi: 10.1038/s41586-025-08908-0 40132641 PMC12333957

[21] Nishimura Y, Shimizu H. Cellular receptors for human enterovirus species a. Front Microbiol. 2012;3:105. doi: 10.3389/fmicb.2012.00105 22470371 PMC3313065

[22] Patel KP, Bergelson JM. Receptors identified for hand, foot and mouth virus. Nat Med. 2009;15(7):728–9. doi: 10.1038/nm0709-728 19584857

[23] Li Z, Li H, Liu X, Zhou J, Gao D, Yang W, et al. Characterisation of a secreted MFSD6-Fc microbody as a decoy receptor for respiratory enterovirus D68. EBioMedicine. 2025;120:105915. doi: 10.1016/j.ebiom.2025.105915 40925193 PMC12452593

[24] Yamayoshi S, Ohka S, Fujii K, Koike S. Functional comparison of SCARB2 and PSGL1 as receptors for enterovirus 71. J Virol. 2013;87(6):3335–47. doi: 10.1128/JVI.02070-12 23302872 PMC3592140

[25] Plevka P, Lim P-Y, Perera R, Cardosa J, Suksatu A, Kuhn RJ, et al. Neutralizing antibodies can initiate genome release from human enterovirus 71. Proc Natl Acad Sci U S A. 2014;111(6):2134–9. doi: 10.1073/pnas.1320624111 24469789 PMC3926013

[26] Kobayashi K, Koike S. Cellular receptors for enterovirus A71. J Biomed Sci. 2020;27(1):23. doi: 10.1186/s12929-020-0615-9 31924205 PMC6954530

[27] Nishimura Y, Shimojima M, Tano Y, Miyamura T, Wakita T, Shimizu H. Human P-selectin glycoprotein ligand-1 is a functional receptor for enterovirus 71. Nat Med. 2009;15(7):794–7. doi: 10.1038/nm.1961 19543284

[28] Yamayoshi S, Fujii K, Koike S. Receptors for enterovirus 71. Emerg Microbes Infect. 2014;3(7):e53. doi: 10.1038/emi.2014.49 26038749 PMC4126179

[29] Zhu Y, Wang L, Shen J. Enterovirus D68 Sequence Variations and Pathogenicity: A Review. Viruses. 2026;18(1):73. doi: 10.3390/v18010073 41600837 PMC12846453

[30] Nishimura Y, Lee H, Hafenstein S, Kataoka C, Wakita T, Bergelson JM, et al. Enterovirus 71 binding to PSGL-1 on leukocytes: VP1-145 acts as a molecular switch to control receptor interaction. PLoS Pathog. 2013;9(7):e1003511. doi: 10.1371/journal.ppat.1003511 23935488 PMC3723564

[31] Li X, Liu Z, Yan X, Tian Y, Liu K, Zhao Y, et al. VP2 residue N142 of coxsackievirus A10 is critical for the interaction with KREMEN1 receptor and neutralizing antibodies and the pathogenicity in mice. PLoS Pathog. 2023;19(10):e1011662. doi: 10.1371/journal.ppat.1011662 37788227 PMC10547193

[32] Klatzmann D, Champagne E, Chamaret S, Gruest J, Guetard D, Hercend T, et al. T-lymphocyte T4 molecule behaves as the receptor for human retrovirus LAV. Nature. 1984;312(5996):767–8. doi: 10.1038/312767a0 6083454

[33] Liu D, Ji Z, Zheng X, Xia H, Yang W, Ge P-F, et al. Enterovirus D68 receptor usage: from static attachment to dynamic entry. J Virol. 2026;100(2):e0194925. doi: 10.1128/jvi.01949-25 41467840 PMC12911884

[34] Zhang X, Shi J, Ye X, Ku Z, Zhang C, Liu Q, et al. Coxsackievirus A16 utilizes cell surface heparan sulfate glycosaminoglycans as its attachment receptor. Emerg Microbes Infect. 2017;6(7):e65. doi: 10.1038/emi.2017.55 28745308 PMC5567171

