# Supplementary material for: Intracellular SCARB2 mimicry overcomes decoy failure to broadly inhibit enteroviruses
Source: PLoS Pathog. 2026 Oct 13;22(10):e1014699. doi: 10.1371/journal.ppat.1014699 (PMC13705605; doi:10.1371/journal.ppat.1014699)
Supplement: Supplementary file 1 [file ppat.1014699.s012.pdf]

Fig. 1C (VP1)

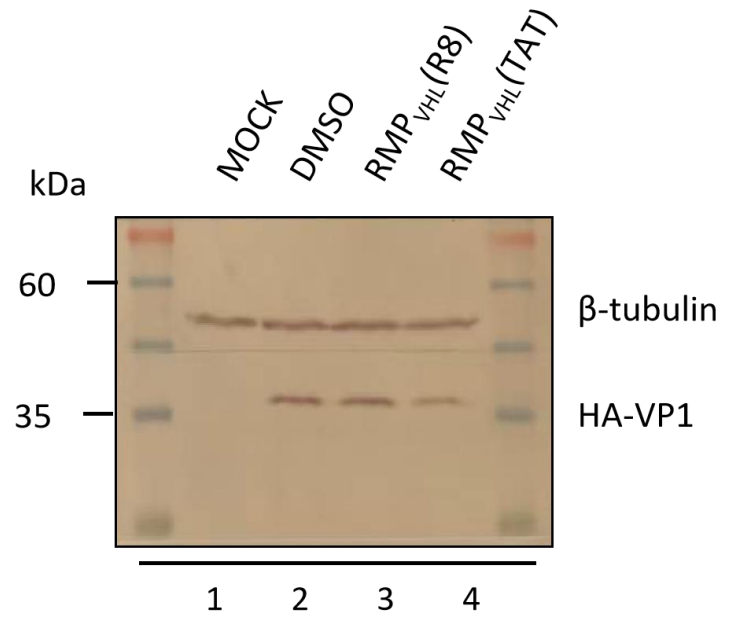

Fig. 1C (VP2)

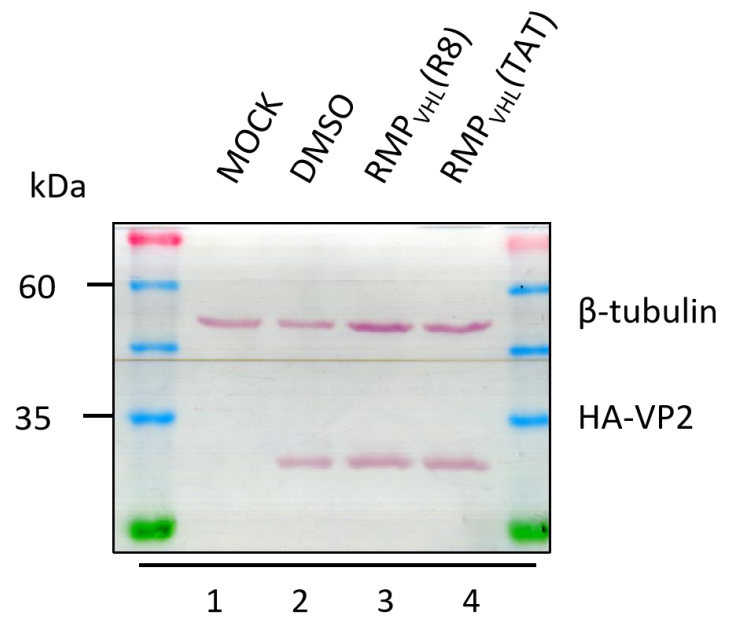

Fig. 1I (RMP$_{VHL}$R8)

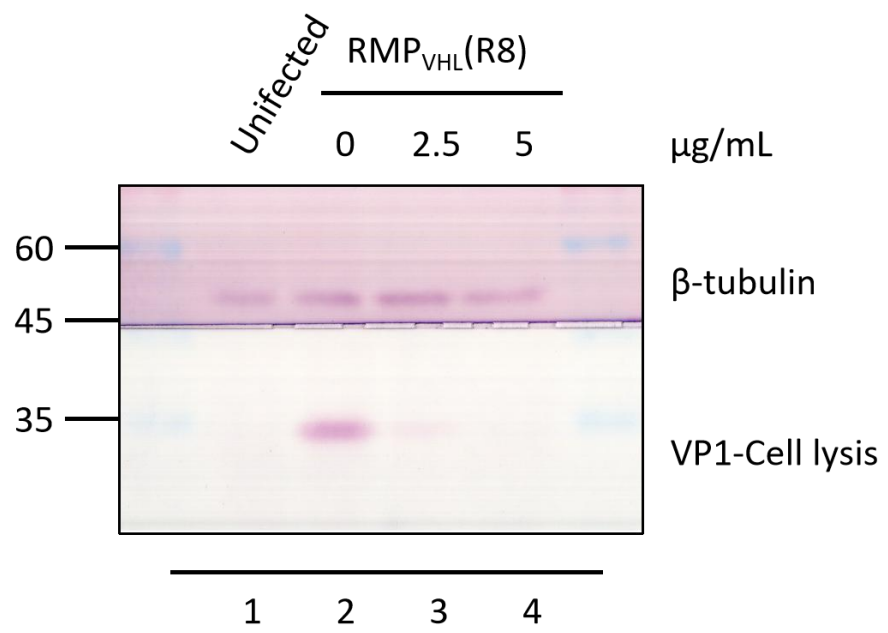

Fig. 1I (RMP$_{VHL}$TAT)

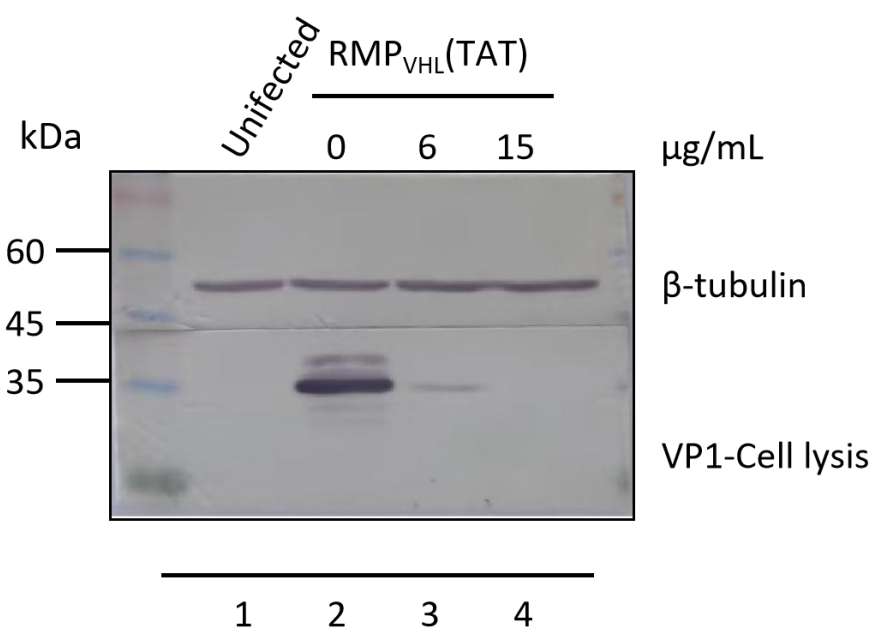

Fig. 2K

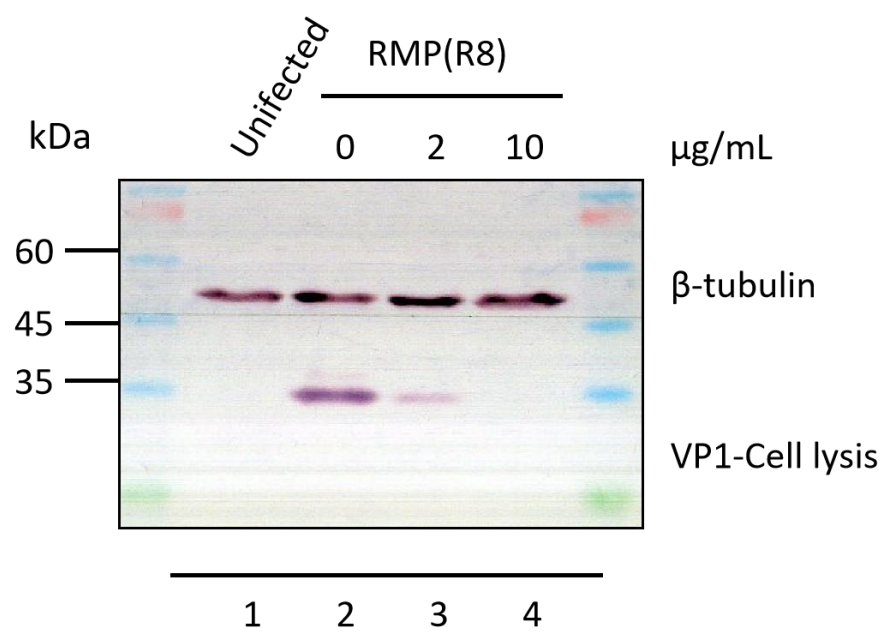

Fig. 2L

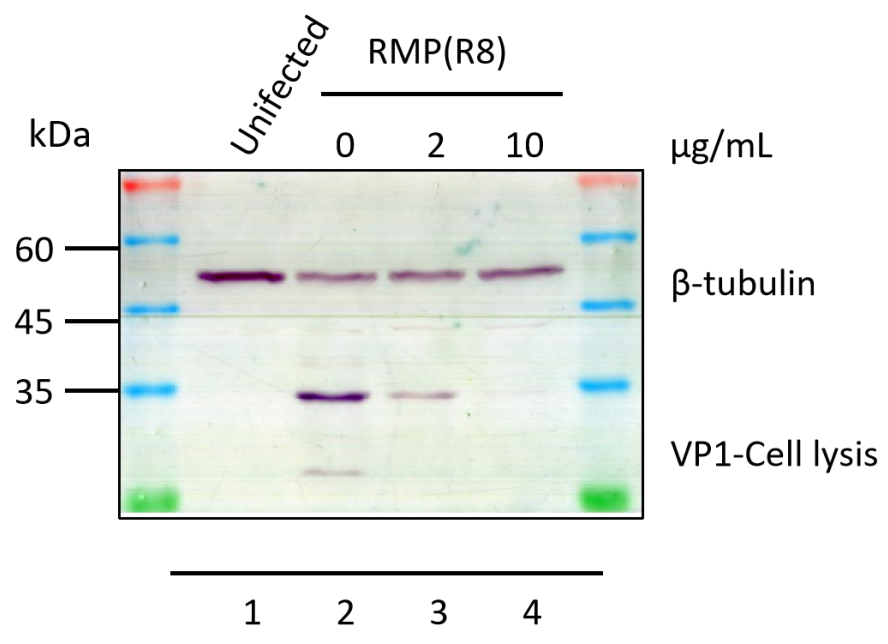

Fig. 2M

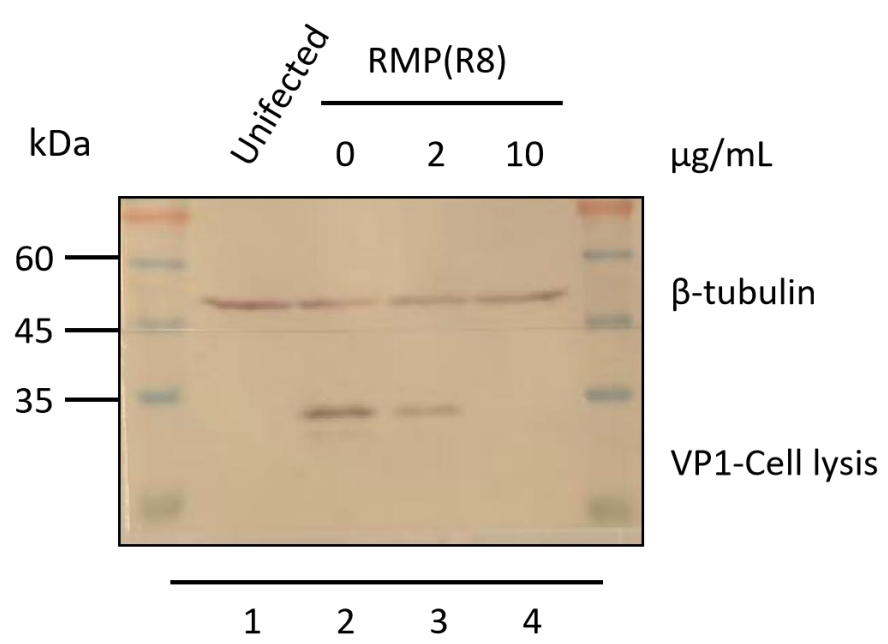

Fig. 3F

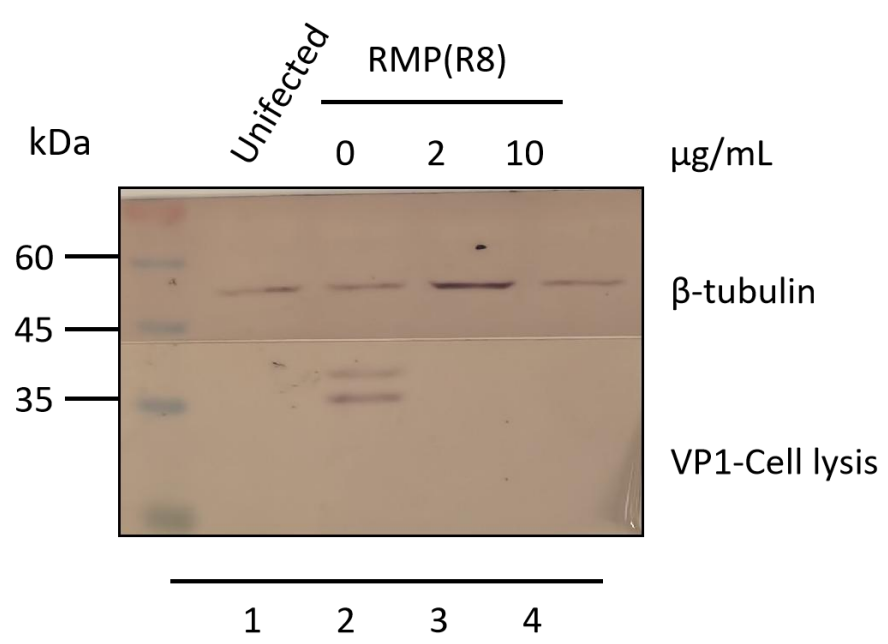

Fig. 3G

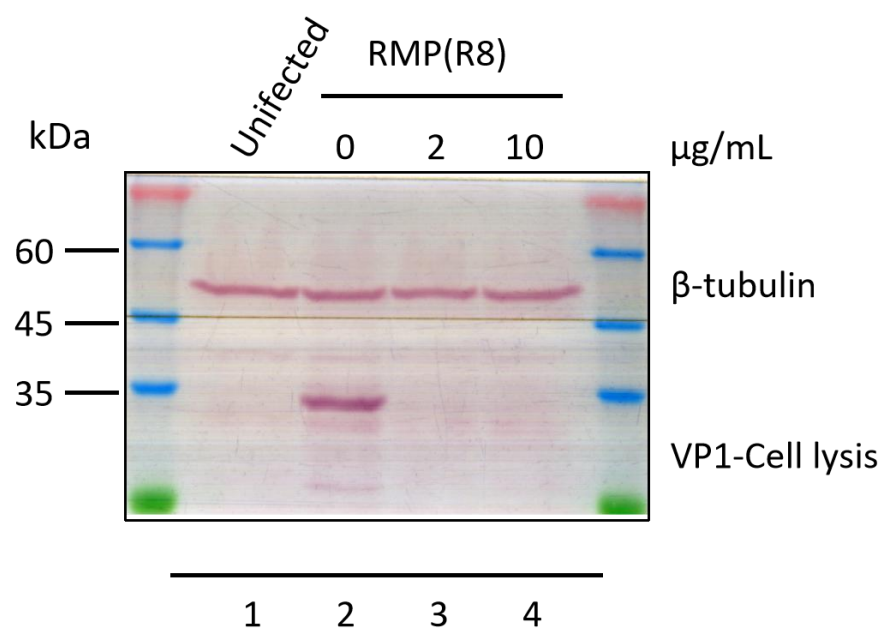

Fig. 3H

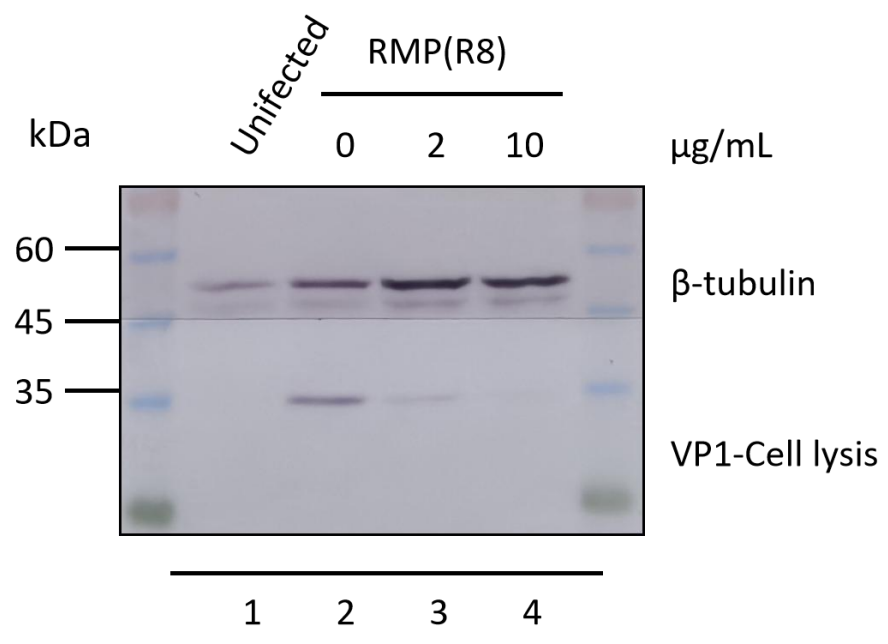

Fig. 3I

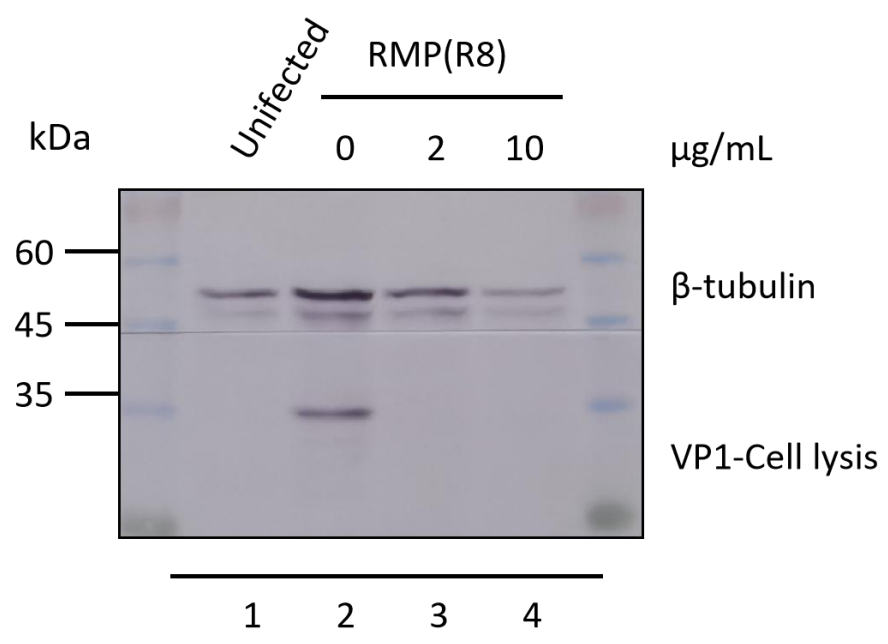

Fig. 4B

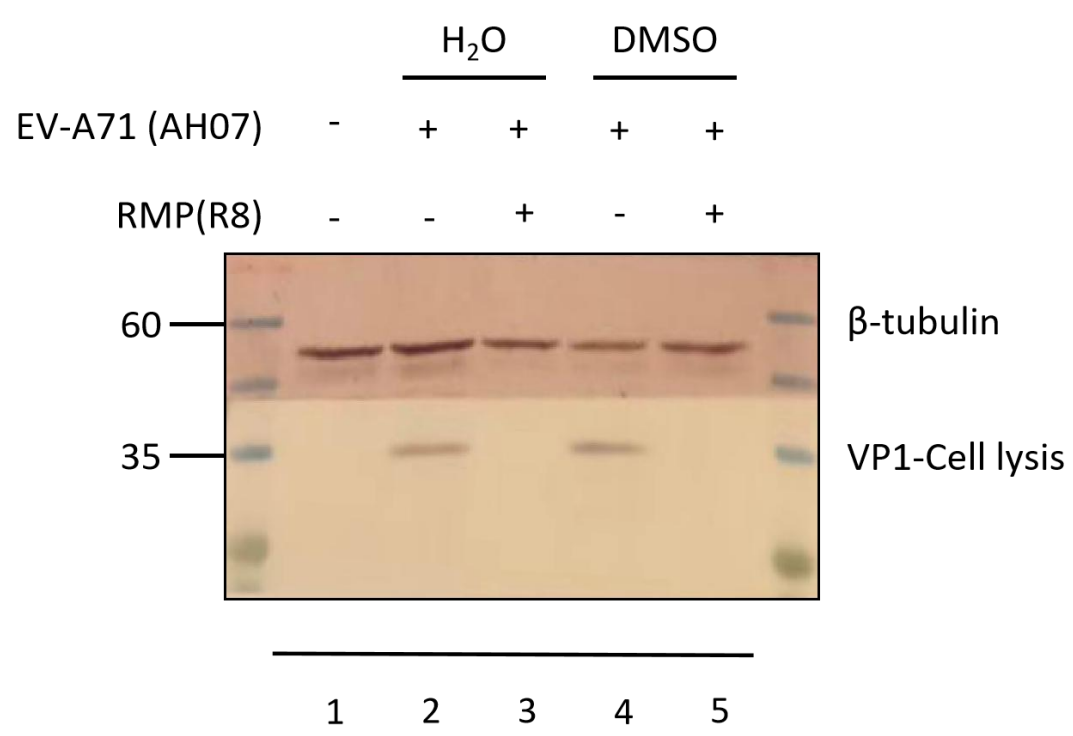

Fig. S1B

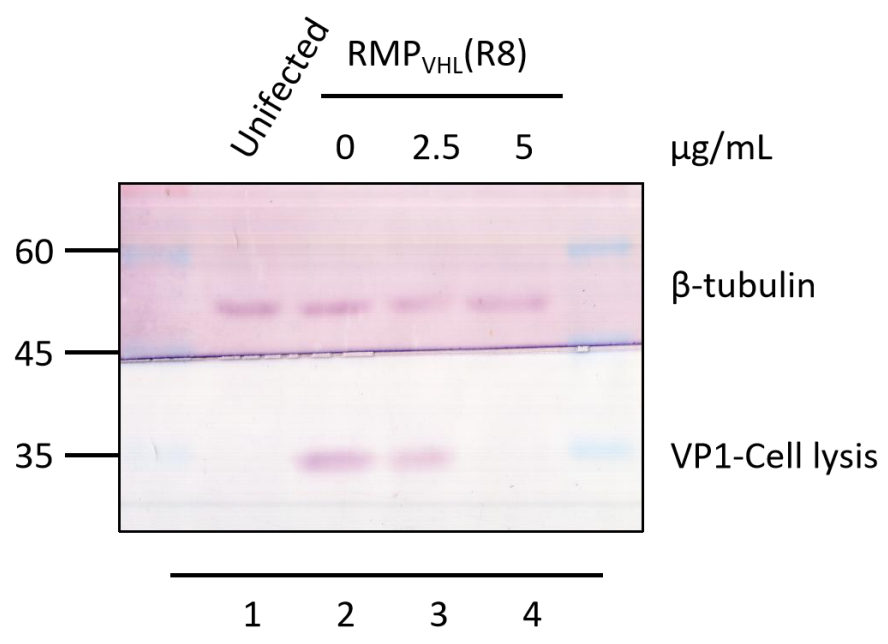

Fig. S1C

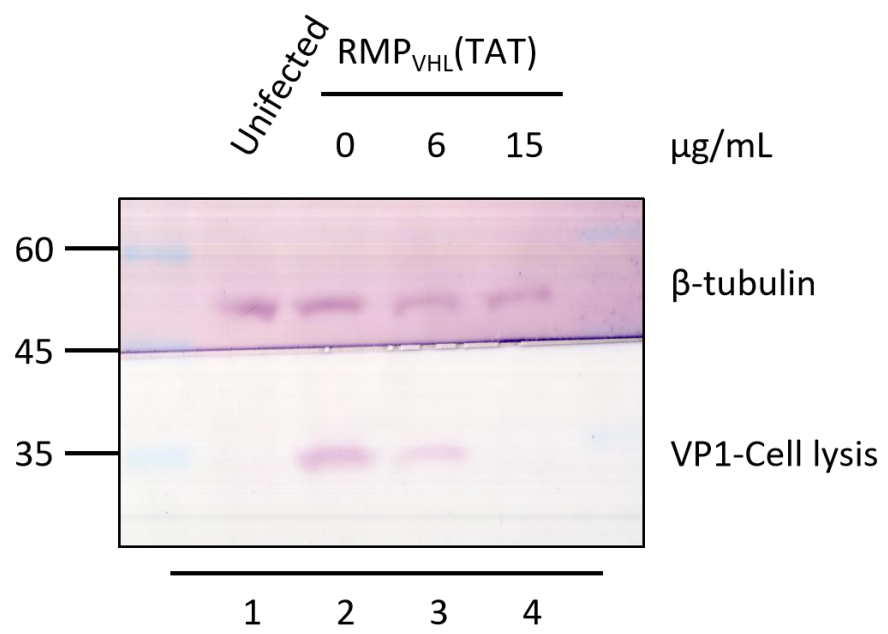

Fig. S2

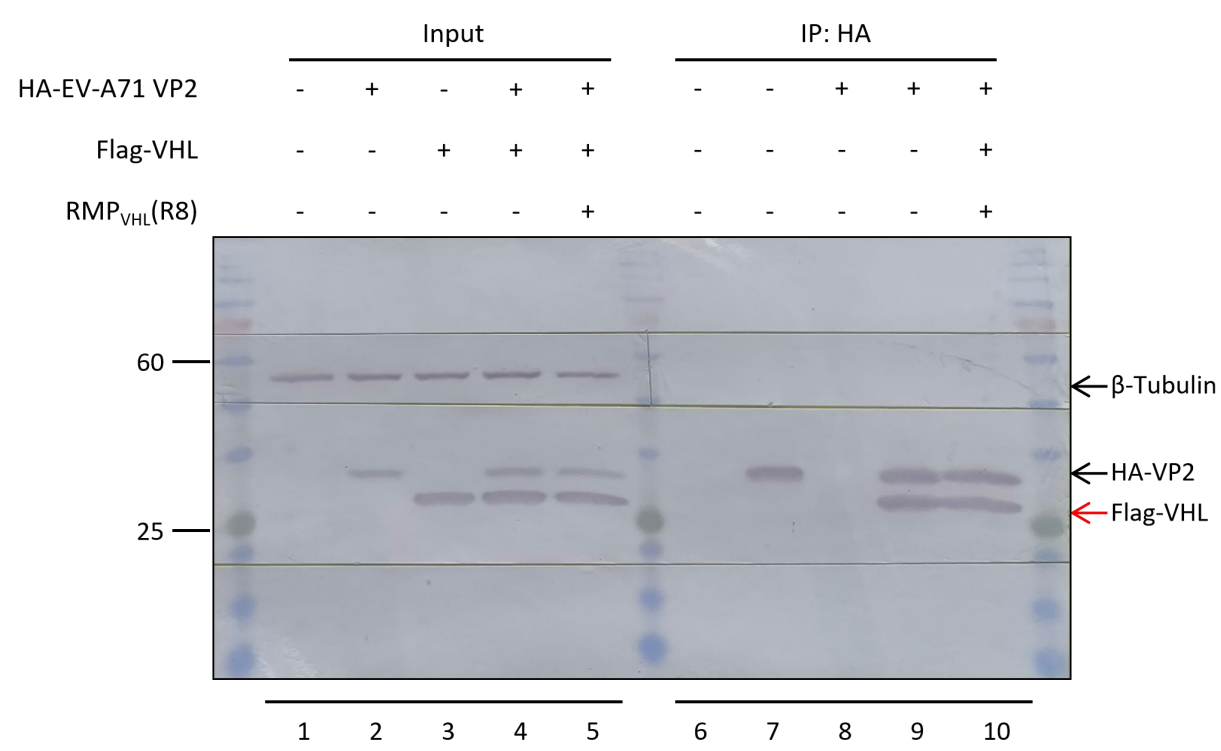

Fig. S5B

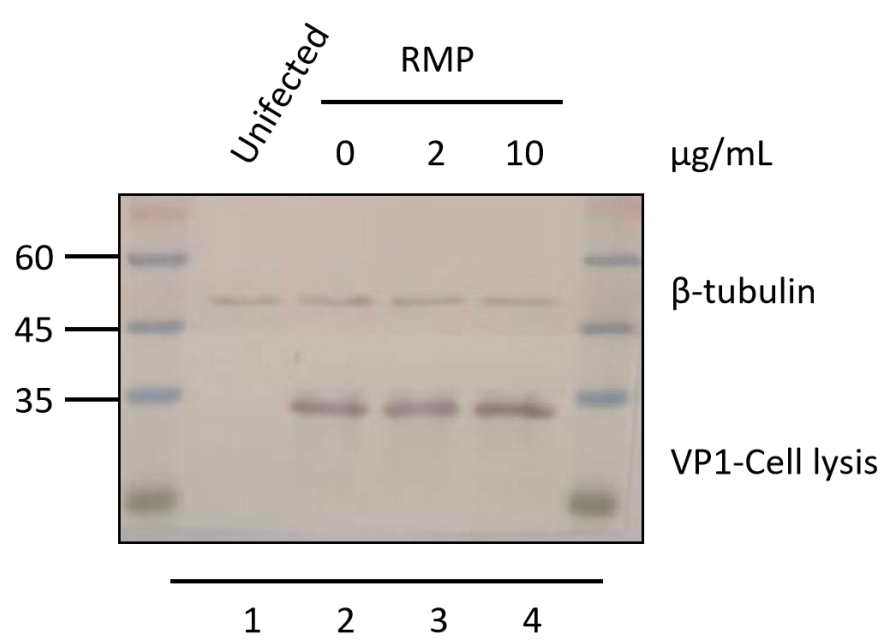

Fig. S5C

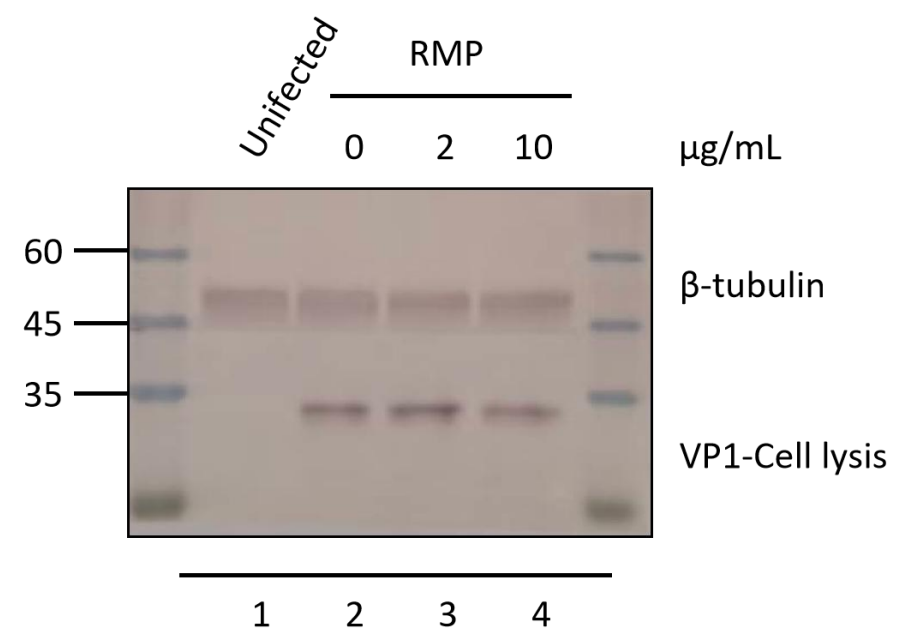

Fig. S6B

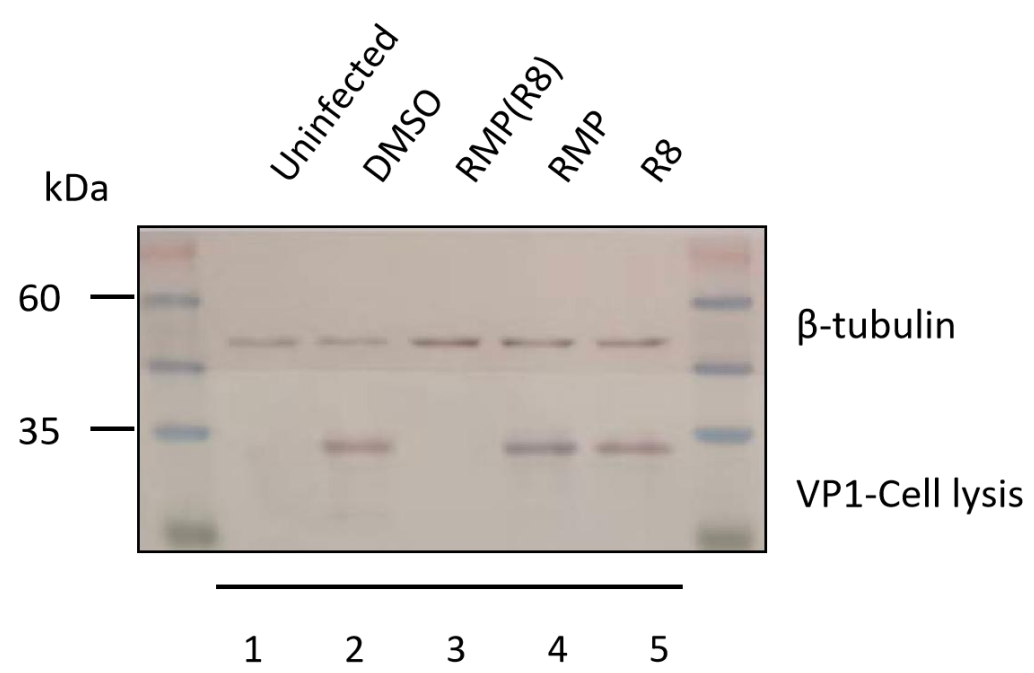

Fig. S7D

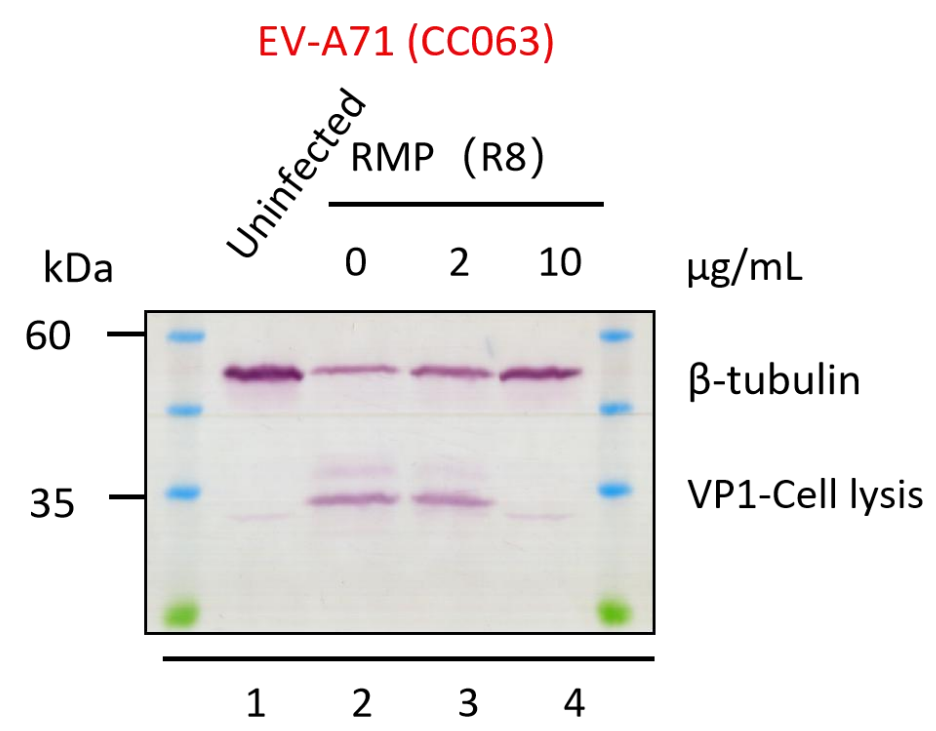

Fig. S7E

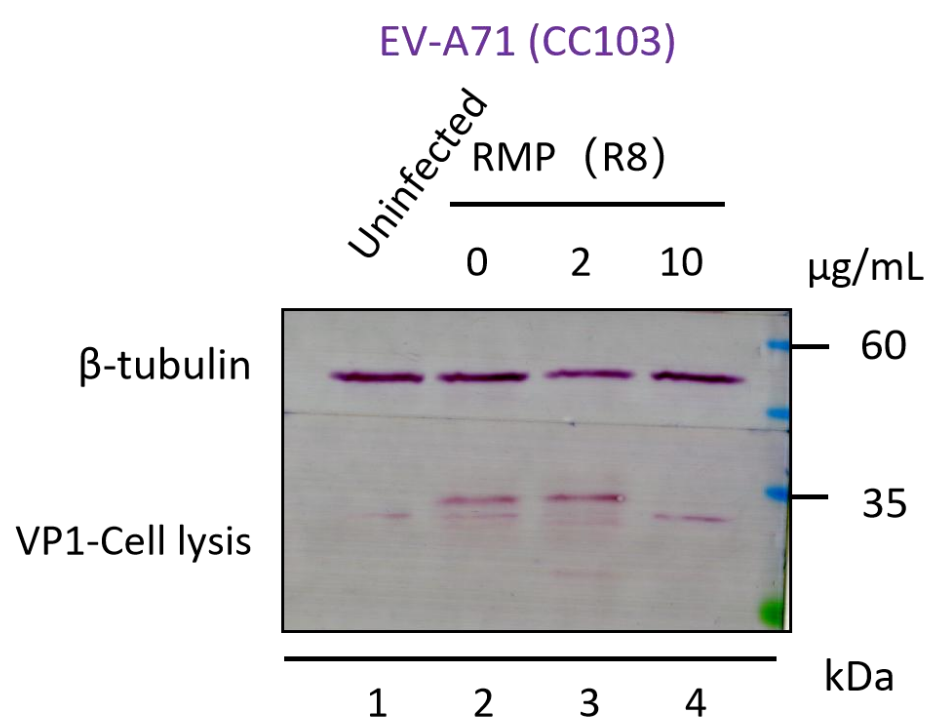

Fig. S11C

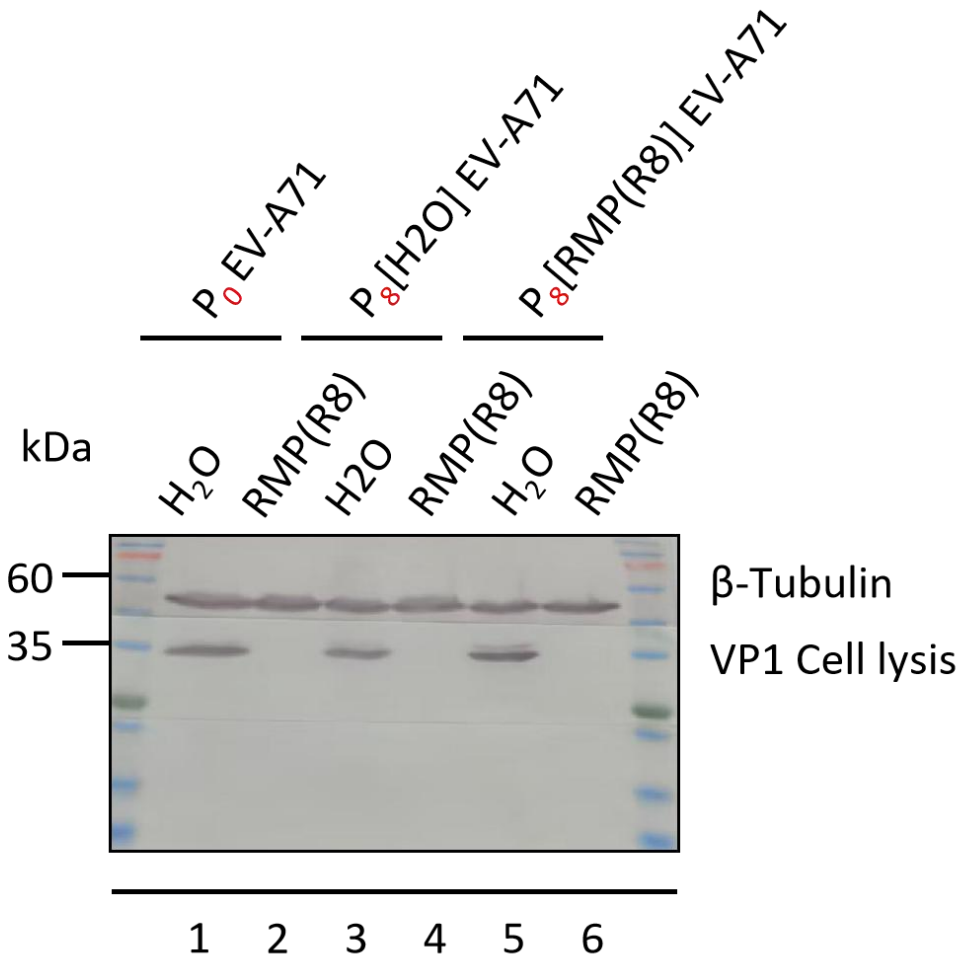

P0 EV-A71
P8[H2O] EV-A71
P8[RMP(R8)] EV-A71
kDa
H2O
RMP(R8)
H2O
RMP(R8)
H2O
RMP(R8)
60
35
β-Tubulin
VP1 Cell lysis
1
2
3
4
5
6
